# NAT10‐mediated N4‐acetylcytidine modification drives RNA splicing of PML to alleviate adipose‐derived stem cell senescence and promote diabetic wound healing

**DOI:** 10.1002/ctm2.70711

**Published:** 2026-06-11

**Authors:** Wuhan Wei, Chanyuan Jiang, Dong Zhu, Xuefeng Han, Xuan Ma, Xinyu Jia, Jiaqi Ling, Rui Zhang, Facheng Li, Ningbei Yin

**Affiliations:** ^1^ Plastic Surgery Hospital Chinese Academy of Medical Sciences and Peking Union Medical College Beijing People's Republic of China

**Keywords:** NAT10, N4‐acetylcytidine, RNA splicing, senescence, stem cells

## Abstract

**Background:**

Cellular senescence of adipose‐derived stem cells (ADSCs) compromises their therapeutic potential in diabetic wound healing. Alternative splicing produces functionally different variants and serves as a critical regulator of senescence. N‐acetyltransferase 10 (NAT10) is known to catalyse N4‐acetylcytidine (ac4C) RNA modification, and ac4C modification has been involved in RNA splicing. Nevertheless, how NAT10 functions in ADSCs remain unexplored. The aim of this study was to investigate the involvement of NAT10 in ADSC senescence and its impact on RNA splicing.

**Methods:**

Senescence was assessed by β‐galactosidase staining, western blot analysis of p21 and p16 and qRT‐PCR detection of senescence‐associated secretory phenotype (SASP) genes. The role of NAT10 in splicing regulation was examined by RT‑PCR.

**Results:**

NAT10 overexpression mitigated ADSC senescence under high‐glucose conditions and augmented the wound repair capability of ADSCs. Mechanistically, NAT10 facilitated ac4C‐dependent AS of the PML transcript, driving a switch from the long isoform (PML‐FL) to the short isoform (PML‐S). PML‐FL accelerated cellular senescence, whereas PML‐S suppressed it. NAT10 recruited SRSF1 to PML pre‐mRNA, leading to ac4C‐SRSF1‐mediated exon skipping and increased PML‐S production. Concurrently, NAT10 reduced the binding of PCBP1 to PML, thereby inhibiting PML‐FL generation.

**Conclusions:**

Our findings uncover a previously unrecognised mechanism by which NAT10 regulates ADSC senescence through ac4C‐dependent alternative splicing and suggest a potential strategy to improve ADSC‐based therapies for diabetic wounds.

**Key points:**

- NAT10 catalyzes ac4C‐dependent alternative splicing of PML pre‐mRNA, shifting the balance from the pro‐senescence PML‐FL isoform to the protective PML‐S isoform.

## Graphical abstract

N‐acetyltransferase 10 (NAT10) catalyses N4‐acetylcytidine (ac4C) modification of PML pre‐mRNA, shifting splicing from the senescence‐promoting PML‐FL to the senescence‐inhibiting PML‐S isoform via SRSF1. This rejuvenates adipose‐derived stem cells (ADSCs) by reducing senescence markers and senescence‐associated secretory phenotype (SASP), thereby accelerating diabetic wound healing.

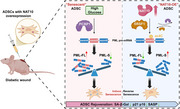

## 1 INTRODUCTION

Diabetic wounds represent one of the most severe complications arising from diabetes and contribute substantially to global disability and mortality burdens.^1^, ^2^ Adipose‐derived stem cells (ADSCs) have been proposed as potential therapeutic candidates to treat diabetic wounds.^3^ However, the high‐glucose (HG) microenvironment in diabetic wounds drives transplanted and resident stem cells into a state of senescence and dysfunction, undermining their potential efficacy.^4^, ^5^ Therefore, clarifying the mechanisms underlying ADSC senescence and discovering new molecular targets are important for improving stem cell‐based treatment strategies for diabetic wounds.

Alternative splicing (AS) is a fundamental regulatory mechanism that expands the functional diversity of cells, specifically by splicing precursor mRNA (pre‐mRNA) to produce various mRNA and protein variants.^6^ Accumulating evidence indicates that AS regulates stem cell properties, including pluripotency, aging, proliferation and differentiation, by producing diverse transcript and protein variants.^7^, ^8^ Targeting RNA splicing defects in stem cells is now recognised as a viable therapeutic strategy. For example, targeting RBM20 mutations in pluripotent stem cells (PSCs) restores AS patterns of genes associated with dilated cardiomyopathy,^9^ and AS can be induced by CRISPR‐mediated cytidine deaminase in PSCs, leading to the restoration of dystrophin function in Duchenne muscular dystrophy.^10^ Moreover, senescent stem cells undergo extensive AS events, and modulating these events can influence stem cell fate, offering potential therapeutic opportunities to reverse cellular senescence.^11^ Thus, identifying novel senescence‐associated aberrant splicing events and clarifying the underlying mechanisms may provide promising strategies for improving ADSC‐based therapies by correcting RNA splicing abnormalities.

N4‐acetylcytidine (ac4C) is a crucial and ubiquitous RNA modification that has been identified in transfer RNA (tRNA),^12^, ^13^ ribosomal RNA (rRNA),^14^ and messenger RNA (mRNA).^15^, ^16^, ^17^ Dysregulated ac4C modification has been increasingly associated with aging‐related diseases, including liver fibrosis^18^ and premature ovarian failure,^19^ highlighting its importance in cellular senescence. N‐acetyltransferase 10 (NAT10), which exhibits both acetyltransferase and RNA‐binding activities, is the only known writer enzyme that catalyses ac4C modification on eukaryotic mRNAs.^20^, ^21^, ^22^ Recent studies have revealed that RNA splicing can be regulated by various RNA modifications. For example, m^6^A methylation has been reported to influence AS in cancer,^23^ and emerging evidence indicates that ac4C modification plays a similar role. NAT10‐mediated ac4C modification has been shown to regulate the splicing of specific transcripts during viral replication and in plant thermosensory flowering.^24^, ^25^ Notably, a recent study demonstrated that NAT10 undergoes liquid–liquid phase separation (LLPS) and interacts with the splicing factor SRSF2, resulting in SRSF2 acetylation and altered splicing of the m6A reader YTHDF1, thereby promoting gastric cancer progression.^26^ This establishes a direct mechanistic link between NAT10's acetyltransferase activity and AS regulation. However, how the NAT10–ac4C axis governs RNA splicing, particularly in senescence‐associated aberrant splicing, remains largely unexplored.

Beyond its epitranscriptomic function, NAT10 is a multifunctional protein involved in diverse biological processes. It localises primarily to the nucleolus and can acetylate both RNA and protein substrates. NAT10 is also a critical regulator of stem cell fate; for example, it maintains human embryonic stem cell pluripotency by stabilising POU5F1 mRNA and promotes lineage differentiation by coupling mRNA acetylation to chromatin signalling.^20^, ^27^ In the context of aging, changes in NAT10 expression can dynamically modulate cellular senescence. NAT10 has recently been shown to drive tubular epithelial cell senescence in acute kidney injury by modulating DDX17^28^ and to drive podocyte senescence in nephropathy models.^29^ Furthermore, NAT10 dysregulation is associated with a range of aging‐related disorders and has been recognised as a driver of cancer progression, influencing proliferation, metastasis and immune evasion.^30^, ^31^ Despite these advances, the involvement of NAT10 in ADSC senescence within the diabetic microenvironment, and its potential to reshape the AS landscape critical for stem cell function and wound repair, remains unknown. Elucidating this relationship may help develop new approaches for improving ADSC‐based therapeutic efficacy in diabetic wound healing.

The promyelocytic leukaemia protein (PML) exerts a dual role in mediating cellular senescence. PML silencing can promote senescence in triple‐negative breast cancer cells,^32^ whereas its overexpression induces senescence in fibroblasts.^33^ This dual regulation may arise from AS of PML pre‐mRNA, generating multiple isoforms with distinct and even opposing effects on senescence.^34^, ^35^ In this study, we demonstrate that, under HG conditions, poly(C)‐binding protein 1 (PCBP1) enhances the generation of the full‑length PML isoform (PML‐FL), thereby promoting ADSC senescence. Conversely, NAT10 catalyses ac4C modification on PML and enhances its binding to SRSF1, which shifts splicing towards the short PML isoform (PML‐S), which alleviates ADSC senescence. In summary, our findings reveal that NAT10‐directed ac4C modification rectifies PML aberrant splicing, offering new insights into strategies for enhancing ADSC‐based therapy in diabetic wound healing.

## 2 METHODS

### 2.1 Isolation of ADSCs

ADSCs were obtained from lipoaspirates of 10 healthy female donors (body mass index [BMI] < 25 kg/m^2^, aged 25–35 years) following a previously described isolation protocol.^36^ All procedures were performed at the Body Contouring and Fat Grafting Center of Plastic Surgery Hospital, Chinese Academy of Medical Sciences. In each independent experiment, ADSCs from a single donor were employed, and replicates were performed with cells from different donors to ensure reproducibility. This study complied with the Declaration of Helsinki. All donors provided written informed consent for the use and analysis of their data. The collection and use of adipose tissues received approval from the Ethics Committee of Plastic Surgery Hospital, Chinese Academy of Medical Sciences (Approval No. EAEC 2021‐I2M‐1‐052).

### 2.2 Plasmid construction and RNA interference assay

For overexpression experiments, plasmids encoding NAT10, PML‐FL, PML‐S or PCBP1 were synthesised by Haixing Biosciences. Small interfering RNAs targeting NAT10, PML‐FL, SRSF1 and PCBP1 were synthesised by GenePharma. Antisense oligonucleotides (ASOs) targeting PML‐FL were purchased from Haixing Biosciences (Table 1). Empty plasmid, scrambled small interfering RNA, and control ASOs were used as negative controls. Lipofectamine Stem Transfection Reagent (Invitrogen) was used for all transfections, following the manufacturer's protocol.

**TABLE 1:** Sequence information for siRNAs.

Gene name	Sense (5′–3′)
Negative control	UUCUCCGAACGUGUCACGUTT
si‐NAT10#1	GCAUUUGGGUACUCCAAUATT
si‐NAT10#2	GCAUUUGGGUACUCCAAUATT
si‐PML‐FL	GAGGAUGUCUCCAAUACAATT
si‐SRSF1	AGGACAUUGAGGACGUGUUTT
si‐PCBP1	GGUGUAAGAUCAAAGAGAUTT
ASO‐Ctrl	UUCUCCGAACGUGUCACGUTT
ASO‐PML‐FL	GGAGAGGAUGUCUCCAAUATT

### 2.3 Cellular senescence assay

The cellular senescence was assessed primarily through β‐galactosidase (β‐gal) activity using β‐gal staining kit (Beyotime). ADSCs were fixed in 4% paraformaldehyde for 30 min at room temperature and then incubated with freshly prepared β‐gal staining solution at 37°C overnight. Positive cells exhibiting blue staining were quantified in five randomly selected fields per well using ImageJ software. In addition, the expression levels of the senescence‐related markers p16 and p21 in ADSCs were detected by western blot assay. The expression of senescence‐associated secretory phenotype (SASP) genes, including IL‐6, IL‐8, IL‐1β, ICAM‐1, CCL2 and TNF‐α, was examined by qRT‐PCR assay.

### 2.4 Western blot

Protein lysates were resolved on 4%–20% SDS‐PAGEs (FuturePAGE, ACE) and electroblotted onto nitrocellulose membranes. Membranes were blocked with 5% nonfat milk for 1 h, then incubated with primary antibodies overnight at 4°C and with secondary antibodies for 2 h at room temperature. The bands were visualised on a Tanon imaging system (Tanon). The antibodies used are detailed in Table 2.

**TABLE 2:** Antibodies used in this study.

Antibodies	Source	Item number
NAT10	Immunoway	YT6611
P21	Proteintech	10355‐1‐AP
P16	Proteintech	10883‐1‐AP
SRSF1	Immunoway	YN5803
PCBP1	Immunoway	YN0118
β‐actin	Proteintech	66009‐1‐Ig
GAPDH	Proteintech	60004‐1‐Ig
IgG mouse	Proteintech	SA00001‐1
ac4C	Proteintech	68498‐1‐Ig
Flag	Proteintech	66008‐4‐Ig
HA	Proteintech	51064‐2‐AP
GST	Proteintech	66001‐2‐Ig

### 2.5 RNA isolation, reverse transcription and qRT‐PCR

Total RNA was extracted from cell samples using TRIzol reagent (Invitrogen) according to the manufacturer's instructions. Complementary DNA (cDNA) was synthesised from total RNA with a reverse transcription kit (Vazyme). Real‐time quantitative PCR (qRT‐PCR) was performed on a LightCycler 480 instrument (Roche) with SYBR Green (Vazyme). Relative mRNA expression levels were determined by qRT‐PCR, and each reaction was independently repeated at least three times. Data were analysed using the 2^−ΔΔCT^ method. Primers were designed with the online Primer3 tool, and the primer sequences are listed in Table 3.

**TABLE 3:** Sequence information for qPCR primers.

Gene name	Sequence
NAT10	F: GGTACAGAACTGAGGCCCAT
	R: TTCAACTCCCTCAGCTCCAG
IL‐6	F: CCTCCAGAACAGATTTGAGAGTAGT
	R: GGGTCAGGGGTGGTTATTGC
IL‐8	F: GACATACTCCAAACCTTTCCACCC
	R: TTCAAAAACTTCTCCACAACCCTC
IL‐1β	F: CGAATCTCCGACCACTACTA
	R: AGCCTCGTTATCCCATGTGT
ICAM‐1	F: AGGTTGAACCCCACAGTCAC
	R: TCTGAGACCTCTGGCTTCGT
CCL2	F: GATCTCAGTGCAGAGGCTCG
	R: TCTGGGGAAAGCTAGGGGAA
TNF‐α	F: AGGACACCATGAGCACTGAAAGC
	R: AAGGAGAAGAGGCTGAGGAACAAG
ACTB	F: CTGGGACGACATGGAGAAAA
	R: AAGGAAGGCTGGAAGAGTGC
PML	F: GATGCCGAAAACTCGTCCTC
	R: GCTCTGCCTGCACTTCTTTT
PML‐FL	F: CAGAGAGAGTGAAGGCCCAG
	R: TTCCCCTCCTCAGACTCCAT
PML‐S	F: GGACCCTATTGACGTTGACC
	R: CTGGGCTGTCGTTGTATTGG
CHEK2	F: GTTTCTGTTGGGACTGCTGG
	R: ACGTGCCTTTGGATCCACTA
CHEK2‐FL	F: GTGGAGAGGTAAAGCTGGCT
	R: TTTGTCAAACAGCTCTCCCC
CHEK2‐S	F: AAGGAAGTTTGCTATTGGTTCAG
	R: TGATGATGCAAGGATGATTTAGC
PML‐ac4C site	F: TATCCAAGAAAGCCAGCCCA
	R: ACTTCCTCTTCTGGGCTGTC
PCBP1	F: ACAACACACCATTTCTCCGC
	R: ATGGTGAGTTCATGGGTGGT
SRSF1	F: GCGGTCTGAAAACAGAGTGG
	R: GCCCATCAACTTTAACCCGG
HNRNPA1	F: GCTCACGGACTGTGTGGTAA
	R: GGCCTTGCATTCATAGCTGC
HNRNPL	F: TACGCAGCCGACAACCAAATA
	R: CTCCGGGAGTCATCCGAGT
HNRNPU	F: AGGAAGTTCTTGCTGGACGG
	R: GGCCCCTTTGGTCCTCTAAC
PML‐exno5	F: GGAGGCAGAGAGAGTGAAGG
	R: ATGGAGAAGGCGTACACTGG
PML‐intron5	F: TTAGTGCCTTCCAGCCATGA
	R: GGGCAACTGGGATCGAAATC
PML pre‐mRNA primer1	F: CCCTATCTTGGCCTGCTACA
	R: GGGCCAGGTTCTTGAGTTTG
PML pre‐mRNA primer2	F: GATGCCGAAAACTCGTCCTC
	R: AACTTGCTTTCCCGGTTCAC

### 2.6 Co‑immunoprecipitation assay

Cells were harvested and lysed with co‑immunoprecipitation (co‑IP) lysis buffer. After centrifugation at 12 000 × *g* for 30 min at 4°C, the supernatants were incubated with anti‐NAT10 antibody or control IgG together with magnetic beads overnight at 4°C. Subsequently, the beads were washed five times with co‐IP wash buffer to remove nonspecific binding. SDS loading buffer was then added to the beads, followed by boiling at 100°C for 30 min to achieve complete denaturation. Finally, protein–protein interactions were assessed by western blot assay.

### 2.7 Remodelin treatment and ac4C dot blot

ADSCs were treated with 10 µM Remodelin (MedChemExpress, HY‑100263) or dimethyl sulfoxide as the vehicle control for 48 h, based on previously reported concentrations.^37^, ^38^ NAT10 inhibition efficiency was validated by ac4C dot blot (Figure S8). Briefly, total RNA was extracted, and equal amounts of RNA (1 µg) were spotted onto a Hybond‑N^+^ membrane. After UV cross‑linking, the membrane was probed with anti‑ac4C antibody, subsequently with an horseradish peroxidase‐conjugated secondary antibody, followed by enhanced chemiluminescence detection. Equal RNA loading was verified by methylene blue staining. Signal intensities were measured using ImageJ and normalised to the methylene blue signal.

### 2.8 RNA‐seq and data analysis

ADSCs transfected with empty vector (Vec) or NAT10‑overexpression vector (NAT10‑OE) were subjected to total RNA extraction, using three independent biological replicates per condition. Sequencing libraries were constructed and run on an Illumina NovaSeq system following the manufacturer's instructions. The raw data have been deposited in the Gene Expression Omnibus (GEO) under accession number GSE326590.

AS events were analysed using rMATS version 3.2.5 and classified into five categories: skipped exon (SE), alternative 3′ splice site (A3E), alternative 5′ splice site (A5E), mutually exclusive exons (MXEs) and retained intron (RI). Only events with an average percent spliced‐in (PSI) value ≥.05 in at least 75% of samples were retained for further analysis. Differentially spliced events were identified using Student's *t*‑test, with statistical significance defined as |ΔPSI| ≥ .1 and *p* ≤ .05.

### 2.9 RNA pull‐down assay

Biotin‐labelled RNA was synthesised from PCR‑amplified DNA templates using T7 RNA polymerase in the presence of biotin‑UTP, and the resulting probe was biotinylated at the 5′ terminus. The purified biotin‐labelled transcripts were incubated with cell lysates at room temperature for 1 h. RNA–protein complexes were then captured with streptavidin‑conjugated magnetic beads (BersinBio), and the bound proteins were analysed by western blot.

### 2.10 RIP and acRIP

RNA‐binding protein immunoprecipitation (RIP) and acetylated RNA immunoprecipitation (acRIP) assays were performed as follows. ADSCs were washed with phosphate‐buffered saline and lysed in 1 mL of RIP lysis buffer supplemented with RNase inhibitor on ice for 1 h. After complete lysis, the lysate was centrifuged at 12 000 × *g* for 30 min at 4°C. Ten percent of the supernatant was collected as the Input and stored at −80°C, while the remaining supernatant was incubated overnight at 4°C with protein A/G‐conjugated magnetic beads (MCE) and the indicated antibodies (anti‐NAT10, anti‐ac4C, anti‐SRSF1 or control IgG). Following incubation, the IP samples were washed with RIP wash buffer to minimise nonspecific binding and subsequently incubated in RIP buffer containing SDS and proteinase K at 55°C for 1 h. Total RNA was then extracted from both the IP samples and the Input using TRIzol reagent (Invitrogen) and analysed by qRT‐PCR. Enrichment was expressed as the ratio of target RNA abundance in the IP relative to the Input.

### 2.11 FISH

Cy3‐labelled probes targeting NAT10 and FITC‐labelled probes targeting PML pre‐mRNA were synthesised from GenePharma. Fluorescence in situ hybridisation (FISH) was performed using the FISH Kit (GenePharma). Images were captured on a confocal laser scanning microscope (Zeiss LSM880).

### 2.12 Diabetic wound models and treatment

The animal experiments were approved by the Animal Care and Ethics Committee of Plastic Surgery Hospital, Chinese Academy of Medical Sciences (Project number: EAEC 2025‑024). The male nude mice aged 6–8 weeks were obtained from Plastic Surgery Hospital of Chinese Academy of Medical Sciences. The diabetic mice were created by receiving an intraperitoneal injection of 150 mg/kg streptozotocin (Sigma). Blood glucose was monitored weekly, and animals with non‐fasting serum glucose level over 16.7 mmol/L for at least 4 weeks were deemed diabetes. After adequate anaesthesia with CO_2_, a full‑thickness excisional wound (1.0 cm in diameter) was created on the back skin of mice.

### 2.13 Measurement of ADSCs survival in the wounds

ADSCs (1 × 10^7^ cells) were prelabelled with the fluorescence dye CM‐Dil (Invitrogen) before injections according to the manufacturer's instructions. Labelling efficiency was verified by fluorescence microscopy 24 h after staining. For in vivo tracking, labelled ADSCs were injected into the wound edge immediately after wound creation. The survival and distribution of CM‐Dil‑labelled ADSCs were assessed using the IVIS 200 imaging system (Xenogen, Caliper Life Sciences), with excitation and emission set to 420 and 480 nm. Images were captured on Days 0 and 3 after injection, and signal intensities were measured by Living Image software (PerkinElmer). Representative images from each group are shown in the corresponding figures.

### 2.14 Assessment of wound healing

A total of 40 diabetic nude mice was randomly allocated to eight groups, with five animals per group. For all in vivo experiments, ADSCs (1 × 10^7^ cells) were injected intradermally around the wound edge immediately after wound creation. Wound closure was monitored on Days 0, 7 and 14 after injection. The wound healing rate was assessed with a percentage using the formula: wound closure index (%) = (1 − unhealed wound area/original wound area) × 100%. A total of 40 mice were used across three independent experiments, with *n* = 5 mice per group.

Experiment 1 assessed NAT10 overexpression. Mice received either control vector‑transfected ADSCs (DM + ADSC‑Vec) or NAT10‑overexpressing ADSCs (DM + ADSC‑NAT10). Experiment 2 assessed PML‑S overexpression. Mice received either control vector‑transfected ADSCs (DM + ADSC‑Vec) or PML‑S‑overexpressing ADSCs (DM + ADSC‑PML‑S). Experiment 3 assessed NAT10 overexpression combined with PML‑FL inhibition. Mice were assigned to four groups: DM + ADSC‑Vec + ASO‑Ctrl, DM + ADSC‑NAT10 + ASO‑Ctrl, DM + ADSC‑Vec + ASO‑PML‑FL and DM + ADSC‑NAT10 + ASO‑PML‑FL.

### 2.15 Hematoxylin and eosin (H&E) and Masson staining

On Day 14 after injection, full‐thickness dorsal wounds were excised together with a 2 mm margin of adjacent normal skin. Harvested skin tissues were fixed in 4% paraformaldehyde, paraffin‐embedded and serially sliced at 3 µm. For each wound, sections were obtained from the central region, defined as the midpoint of the wound, to ensure consistent comparisons across groups. The sections were stained using the H&E Staining Kit (Servicebio, G1076) and the Masson Stain Kit (Servicebio, G1006), according to the manufacturer's protocol. Micrographs were captured using an Olympus light microscope.

### 2.16 Statistical analysis

Statistical analysis was carried out using GraphPad Prism (version 9.4.1). Results are expressed as mean ± SD. An unpaired Student's *t*‑test was employed for two‑group comparisons, while one‑way ANOVA followed by Bonferroni or Dunnett post hoc correction was used for multiple‑group comparisons. A *p* value <.05 was considered statistically significant. Western blot, qRT‑PCR and all other experiments were independently repeated at least three times, and representative data are presented.

## 3 RESULTS

### 3.1 NAT10 attenuates ADSC senescence and enhances the reparative capacity of ADSCs in diabetic wounds

The diabetic milieu exerts detrimental effects on ADSCs, and these cells exhibit increased senescence when cultured under HG conditions.^39^ Exposure to HG markedly elevated the percentage of SA‑β‑gal‑positive ADSCs (Figure S1A,B). In addition, HG treatment upregulated the typical senescence markers p21 and p16 and increased the expression of multiple SASP genes (Figure S1D,E).

**FIGURE 1:**
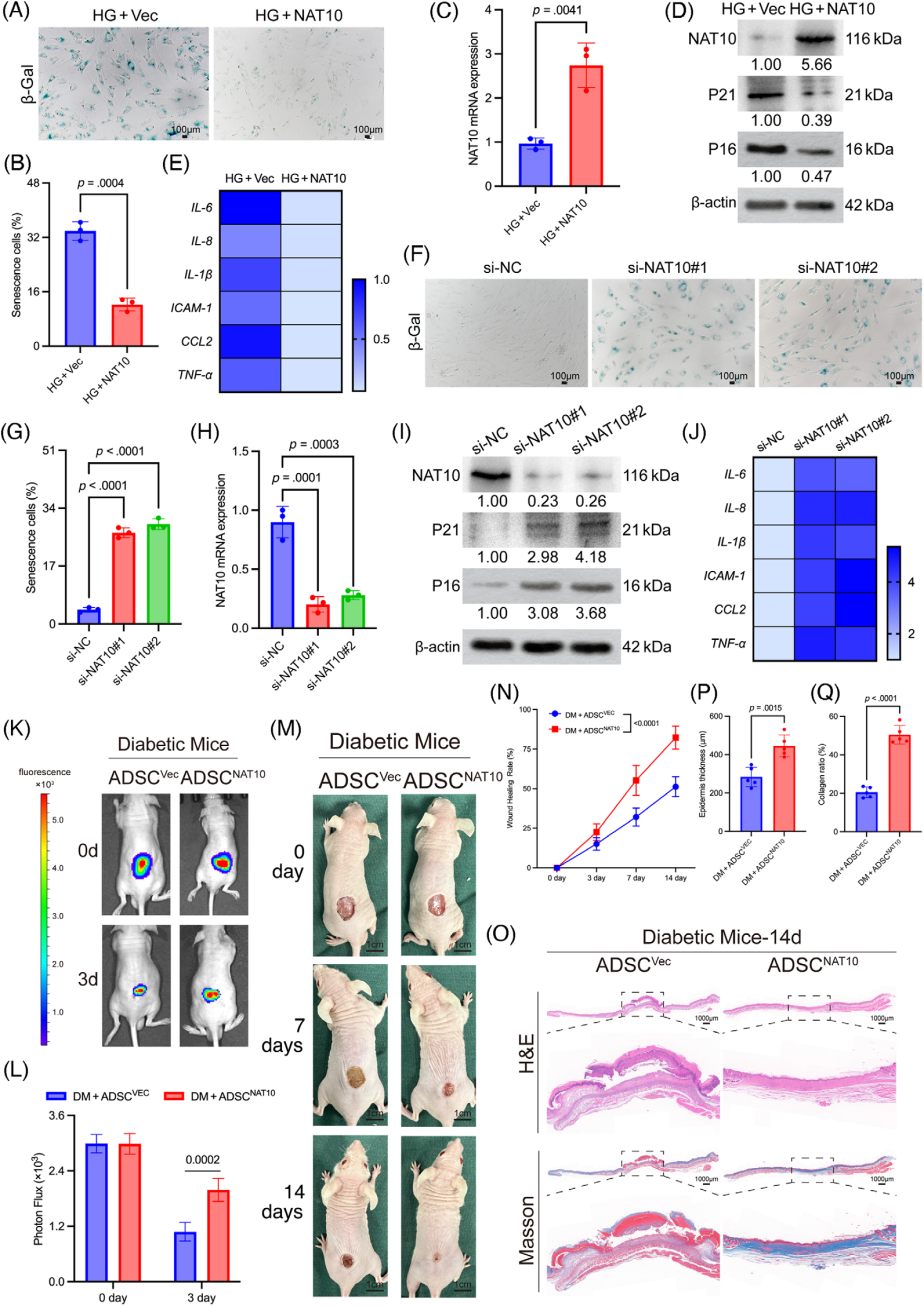
N‐acetyltransferase 10 (NAT10) significantly reduces adipose‐derived stem cell (ADSC) senescence in a high‐glucose environment and enhances ADSC repairability in diabetic wounds. (A, B) β‐Galactosidase (β‐Gal) staining of ADSCs overexpressing NAT10 or vector (Vec) under high glucose (HG). Scale bar, 100 µm. (C) qRT‐PCR analysis of NAT10 expression. (D) Western blot of NAT10, p21 and p16 in ADSCs under HG. (E) Heatmap of SASP gene expression (IL‑6, IL‑8, IL‑1β, ICAM‑1, CCL2, TNF‑α) by qRT‑PCR. (F, G) β‐Gal staining of ADSC transfected with si‐NAT10 or si‑NC. Scale bar, 100 µm. (H) qRT‑PCR of NAT10 mRNA after si‑NAT10. (I) Western blot of NAT10, p21, p16 after NAT10 knockdown. (J) Heatmap of SASP expression after NAT10 knockdown. (K–O) ADSCs overexpressing NAT10 or Vec were injected into diabetic mouse wounds. (K, L) Distribution of CM‐Dil‐labelled ADSCs. (M) Gross wound appearance at Days 0, 7 and 14. Scale bar, 1 cm. (N) Quantification of wound healing rate. (O) Hematoxylin and eosin (H&E) and Masson staining at Day 14. Scale bar, 1000 µm. (P) Quantification of epidermis thickness in each group at Day 14. (Q) Quantification of collagen deposition in each group at Day 14. Data are shown as means ± SD (*n* = 3 independent experiments for in vitro; *n* = 5 mice per group for in vivo). *p* values were calculated using two‑tailed unpaired Student's *t*‑test (two groups) or one‑way ANOVA with Dunnett post hoc test (multiple groups). Exact *p* values are indicated in the graphs.

NAT10 has been reported to regulate cellular senescence and influence stem cell fate determination.^40^, ^41^ We therefore examined NAT10 levels in HG‑treated ADSCs and found that both NAT10 mRNA and protein were significantly decreased (Figure S1C,D). To determine whether NAT10 regulates ADSC senescence, we overexpressed NAT10 and observed that it significantly attenuated HG‑induced senescence (Figures 1A–E and S2A,B). Conversely, NAT10 silencing markedly promoted ADSC senescence (Figures 1F–J and S2C,D). We further evaluated the contribution of NAT10 to ADSC‑mediated tissue repair using diabetic mouse models. NAT10‐overexpressing ADSCs exhibited greater survival in the wound bed than vector‐transfected ADSCs (Figure 1K,L). We then assessed wound healing in diabetic mice and found that NAT10 enhanced the reparative capacity of ADSCs, resulting in a significantly higher wound closure rate (Figure 1M,N). Moreover, NAT10‐overexpressing ADSCs promoted skin regeneration and increased collagen fibre deposition in diabetic wounds (Figure 1O–Q). Together, these findings reveal that NAT10 attenuates ADSC senescence and enhances their ability to promote diabetic wound healing.

### 3.2 NAT10 modulates PML alternative splicing in ADSCs

**FIGURE 2:**
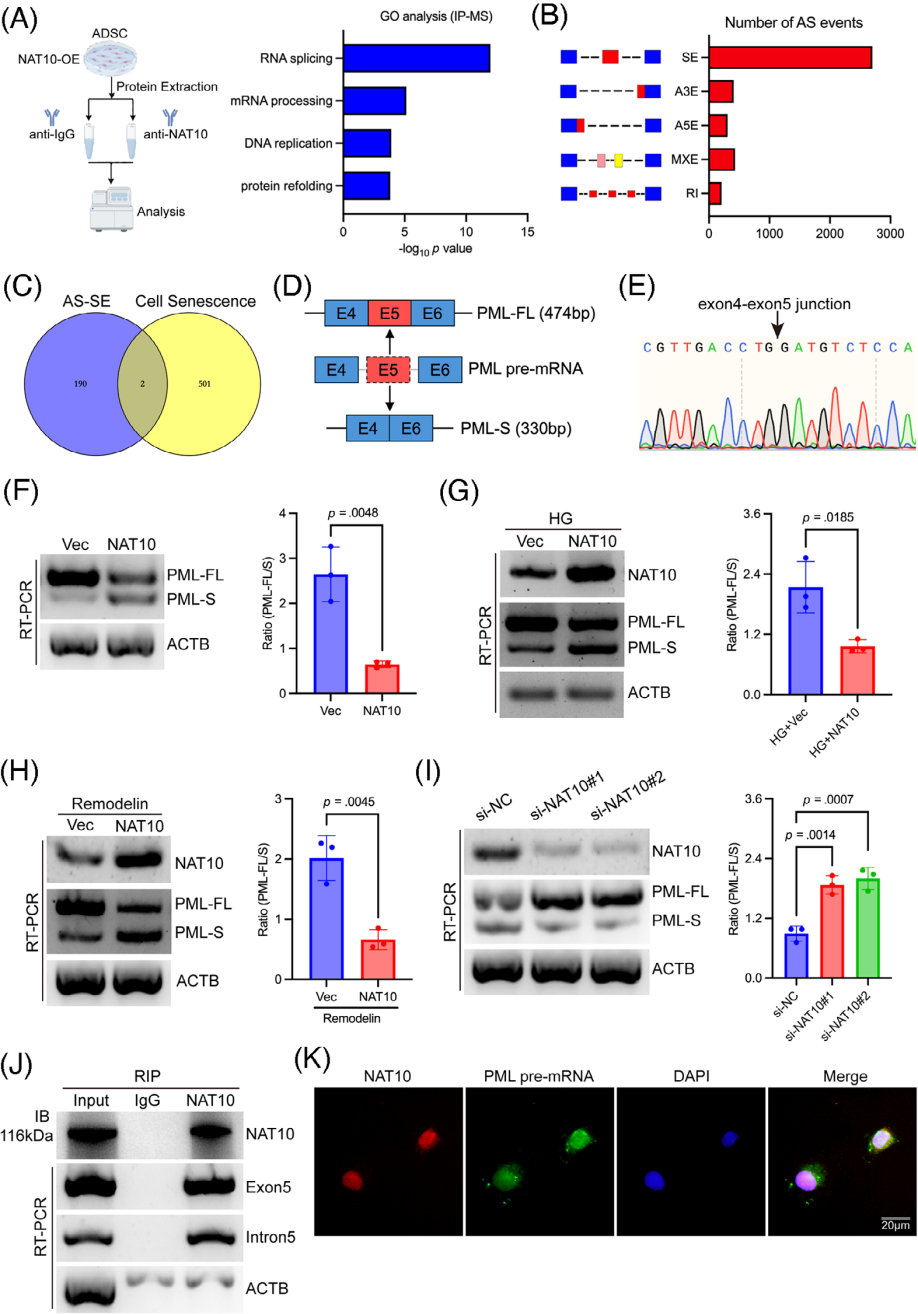
N‐acetyltransferase 10 (NAT10) modulates PML alternative RNA splicing. (A) Gene Ontology (GO) analysis of NAT10‑interacting proteins identified by co‑immunoprecipitation (co‑IP)/MS. (B) Quantification of alternative splicing events affected by NAT10 overexpression (SE, A3E, A5E, MXE, RI). (C) Overlap of NAT10‐regulated SE events with senescence‑related genes. CHEK2 and PML are highlighted. (D) Schematic of PML pre‑mRNA and its isoforms: PML‑FL (exons 4–6) and PML‑S (exon 5 skipped). (E) Sanger sequencing confirming the exon 4‑exon 5 junction. (F) RT‑PCR of PML splicing in NAT10‑OE ADSCs. (G) qRT‑PCR of PML‑FL and PML‑S under HG with NAT10‑OE. (H) RT‑PCR of PML splicing after Remodelin (NAT10 inhibitor, 10 µM, 48 h) treatment in NAT10‑OE ADSCs. (I) qRT‑PCR of PML‑FL and PML‑S after si‑NAT10. (J) RIP assay showing NAT10 binding to PML pre‑mRNA. (K) RNA‑fluorescence in situ hybridisation (FISH; PML pre‑mRNA, green) and immunofluorescence (NAT10, red) with DAPI (blue). Scale bar, 20 µm. Data are shown as means ± SD (*n* = 3). *p* values were calculated using two‑tailed unpaired Student's *t*‑test or one‑way ANOVA with Dunnett post hoc test. Exact *p* values are indicated in the graphs.

We next explored the novel function of NAT10 overexpression in ADSCs. Functional clustering of the NAT10‐interacting proteins by GO analysis revealed that regulation of RNA AS was among the top‐ranked pathways, suggesting that NAT10 may regulate ADSC senescence by modulating RNA splicing (Figure 2A).

To determine mRNA splicing changes upon NAT10 overexpression in ADSCs, we performed RNA‑seq and identified 4056 differential AS events, including SE, A3E, A5E, MXE and RI (Figure 2B). SE events were the most abundant, representing 66.57% of all events. Reactome analysis of these SE events revealed a marked enrichment of categories associated with cellular senescence and RNA splicing (Figure S3A). To pinpoint the targets through which NAT10 regulates RNA splicing and senescence, we intersected the NAT10‑associated SE events with a senescence database.^42^ This analysis identified checkpoint kinase 2 (CHEK2) and PML as candidate targets regulated by NAT10 (Figure 2C). RT‐PCR confirmed that PML splicing was indeed significantly regulated by NAT10 (Figures 2E and S3B).

PML is a tumour suppressor gene whose transcript undergoes AS to generate different isoforms.^43^, ^44^ However, whether NAT10 regulates PML splicing was unknown. We found that exon 5 skipping of PML pre‑mRNA yields PML‑S, whereas exon 5 inclusion produces PML‑FL (Figure 2D). Under HG conditions, NAT10 overexpression in ADSCs led to elevated PML‑S and reduced PML‑FL levels, as assessed by RT‑PCR (Figure 2F). To explore how NAT10 contributes to this splicing switch, we treated ADSCs with Remodelin to suppress endogenous NAT10 activity. RT‐PCR analysis showed that, after NAT10 inhibition, NAT10 overexpression increased PML‐S levels and decreased PML‐FL levels (Figure 2G). Moreover, RT‐PCR confirmed that NAT10 downregulation promoted PML exon inclusion in ADSCs (Figure 2H).

We then performed RIP assays to explore whether NAT10 binds to PML pre‐mRNA. The results showed that NAT10 protein bound both the exon and intron sequences of PML pre‐mRNA in ADSCs (Figure 2I). Consistently, RNA FISH assays demonstrated that NAT10 protein co‐localised with PML pre‐mRNA in the nucleus (Figure 2J). Taken together, these data indicate that NAT10 regulates PML RNA splicing, driving a shift from the PML‐FL isoform towards the PML‐S isoform in ADSCs.

### 3.3 PML‐FL and PML‐S exert opposing effects on ADSC senescence

**FIGURE 3:**
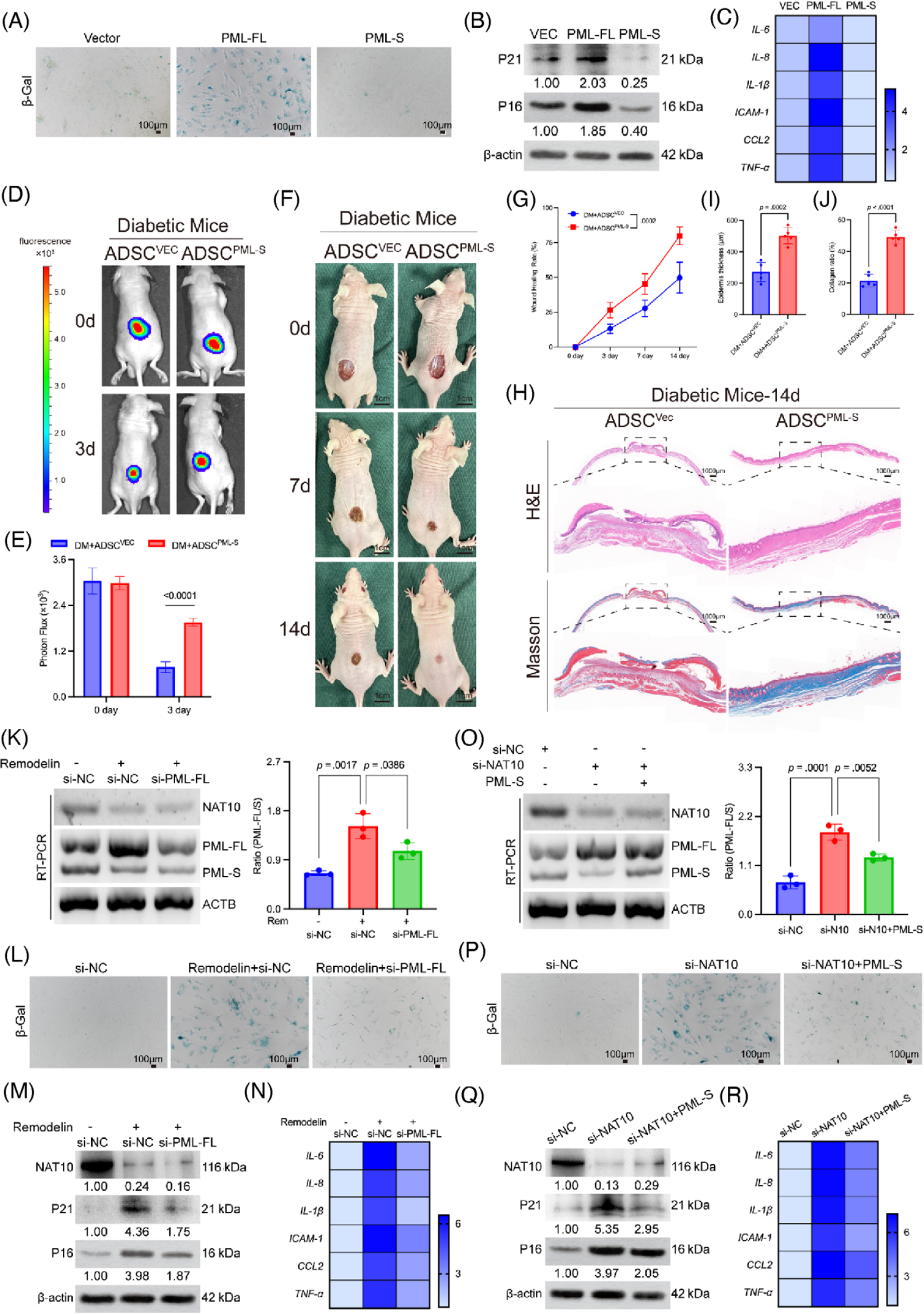
PML‐FL and PML‐S have antithetical functions in adipose‐derived stem cell (ADSC) senescence. (A) β‐Galactosidase (β‑Gal) staining of ADSCs overexpressing Vec, PML‑FL or PML‑S. Scale bar, 100 µm. (B) Expression of NAT10, p21 and p16 was detected by western blot. (C) qRT‑PCR of SASP genes (IL‑6, IL‑8, IL‑1β, ICAM‑1, CCL2, TNF‑α). (D–H) ADSCs overexpressing PML‑S or Vec were injected into diabetic mouse wounds. (D, E) Distribution of CM‐Dil‐labelled ADSCs. (F) Gross wound appearance at Days 0, 7, 14. Scale bar, 1 cm. (G) Quantification of wound healing rate. (H) Hematoxylin and eosin (H&E) and Masson staining at Day 14. Scale bar, 1000 µm. (I) Quantification of epidermis thickness in each group at Day 14. (J) Quantification of collagen deposition in each group at Day 14. (K–R) ADSCs were transfected with si‐PML‐FL with or without Rem (Remodelin, 10 µM, 48 h) (K–N) or transfected with si‐NAT10 and PML‐S overexpression plasmid (O–R). (K, O) RT‑PCR of NAT10 and PML splicing. (L, P) β‐Gal staining assays were conducted to examine cell senescence. Scale bar, 100 µm. (M, Q) Western blot of NAT10, p21 and p16. (N, R) qRT‐PCR of SASP genes in ADSC. Data are shown as means ± SD (*n* = 3 for in vitro; *n* = 5 mice per group for in vivo). *p* values were calculated using two‑tailed unpaired Student's *t*‑test for two‑group comparisons. For multiple‑group comparisons, one‑way ANOVA was performed with Dunnett post hoc test when comparing multiple treatment groups against a single control, or with Bonferroni post hoc test for all pairwise comparisons. Exact *p* values are indicated in the graphs.

PML has been reported to contribute to the regulation of cellular senescence.^45^, ^46^ To investigate the individual contributions of PML‐FL and PML‐S, we overexpressed each isoform in ADSCs. β‐Gal staining, western blot of senescence markers and qRT‐PCR of SASP genes demonstrated that PML‐FL overexpression promoted ADSC senescence, whereas PML‐S overexpression significantly suppressed ADSC senescence (Figures 3A–C and S4A–C). We also evaluated the reparative capacity of ADSCs expressing these two PML isoforms in a diabetic wound model. ADSCs overexpressing PML‐S exhibited enhanced survival in the wound bed, accelerated wound healing, promoted skin regeneration and increased collagen deposition compared with control ADSCs (Figure 3D–J). Moreover, depletion of PML‐FL or overexpression of PML‐S partially reversed the cellular senescence in NAT10‐knockdown cells (Figures 3K–R and S4D–I). Collectively, these observations suggest that PML‑FL and PML‑S differentially regulate senescence, with PML‑S functioning as a protective isoform that suppresses ADSC senescence and boosts regenerative outcomes in diabetic wound healing.

### 3.4 NAT10 promotes ac4C‐SRSF1‐mediated PML exon skipping

**FIGURE 4:**
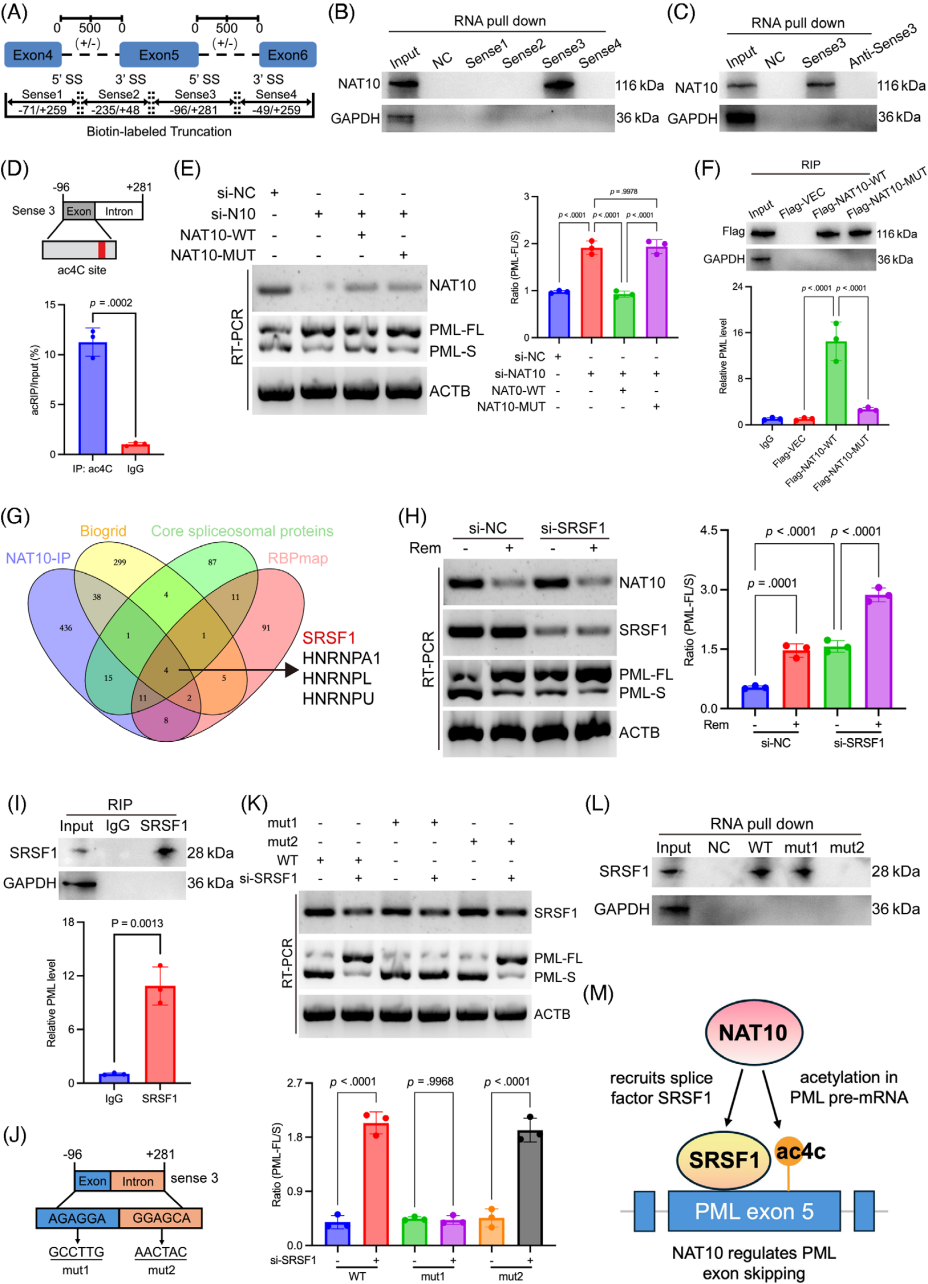
NAT10 recruits SRSF1 to mediate PML exon skipping through ac4C modification. (A) Schematic of biotin‑labelled PML truncations used for RNA pull‑down. (B, C) RNA pull‑down assays detecting NAT10 binding to sense (b) and antisense (c) RNA fragments. (D) acRIP‑qPCR showing ac4C enrichment on the sense 3 region of PML pre‑mRNA. (E) RT‑PCR of PML splicing in NAT10‑depleted ADSCs reconstituted with NAT10‑WT or NAT10‑MUT (ac4C‑binding mutant). (F) RIP‑qPCR measuring binding of Flag‑tagged NAT10‑WT or NAT10‑MUT to PML pre‑mRNA. (G) Venn diagram of overlapping proteins from Biogrid NAT10 interactome, co‑immunoprecipitation (co‑IP)/MS, core spliceosome and splicing factor databases; four factors (including SRSF1) are identified. (H) RT‑PCR of PML splicing after SRSF1 knockdown with or without Remodelin. (I) RIP‑qPCR confirming SRSF1 binding to PML pre‑mRNA. (J) Schematic of two predicted SRSF1 binding sites (mut1, mut2) in the sense 3 region. (K) RT‑PCR of PML minigene reporters (WT, mut1, mut2) in ADSCs transfected with si‑NC or si‑SRSF1. (L) RNA pull‑down using biotin‑labelled WT or mutant probes. SRSF1 binding detected by western blot. (M) Graphical summary illustrating the mechanism by which NAT10 regulates ac4C modification and recruits SRSF1 in RNA splicing of PML. Data are shown as means ± SD (*n* = 3). *p* values were calculated using one‑way ANOVA with Bonferroni post hoc test. Exact *p* values are indicated in the graphs.

To explore how NAT10 regulates PML AS, we identified the region of PML pre‑mRNA that potentially interacts with NAT10. The PML transcript was divided into four fragments, each of which was transcribed in vitro and labelled with biotin at the 5′ terminus (Figure 4A). RNA pull‐down assays revealed that the Sense 3 (−96/+281) specifically bound to NAT10 protein (Figure 4B,C). To further characterise the sense 3 region, we used PACES^16^ to predict potential ac4C modification sites. The results showed that predicted RNA acetylation site was highly enriched in this region (Figure S5A). The predicted acetylation site was validated by acRIP‐qPCR, demonstrating that this site could be acetylated (Figure 4D).

To validate whether NAT10 regulates PML splicing through ac4C recognition, we reconstituted NAT10‐WT (ac4C binding region wild‐type) and NAT10‐MUT (ac4C binding region mutant) in NAT10‐depleted ADSCs. Re‐expression of NAT10‐WT, but not NAT10‐MUT, rescued NAT10‐knockdown‐mediated PML exon 5 inclusion (Figure 4E). Furthermore, RIP assays confirmed that NAT10‐WT, but not NAT10‐MUT, bound PML mRNA (Figure 4F). These data indicate that NAT10 regulates aberrant PML RNA splicing through recognition of ac4C in the sense 3 region of PML pre‐mRNA.

RBPmap was employed to predict candidate splicing factors interacting with PML pre‑mRNA, with the aim of identifying the one that cooperates with NAT10 to regulate PML exon skipping.^47^ We then overlapped these splicing factors with NAT10 co‐IP proteins, core spliceosomal proteins,^48^ and NAT10‐interacting proteins from the Biogrid database (https://thebiogrid.org/). This analysis identified multiple splicing factors that could potentially interact with NAT10, including SRSF1, HNRNPA1, HNRNPL and HNRNPU (Figure 4G). To determine which splice factor is responsible for NAT10‐regulated PML splicing, we transfected ADSCs with siRNA targeting individual splicing factors, with or without Remodelin treatment. RT‐PCR analysis revealed that only SRSF1 depletion significantly inhibited PML exon 5 skipping (Figures 4H and S5B–D). After simultaneous depletion of NAT10 and SRSF1, the PML‐FL/PML‐S ratio was higher than that in single‐knockdown ADSCs (Figure 4H). Furthermore, RIP assays demonstrated that SRSF1 protein interacted with PML pre‐mRNA (Figure 4I).

We then validated two SRSF1‑binding motifs predicted by RBPmap, both located in the sense 3 region of PML pre‑mRNA (Figure 4J). PML minigene reporter assays with targeted mutations showed that the SRSF1‑mediated splicing switch required the first predicted site, as mutating this site abolished the effect of SRSF1 depletion on PML exon inclusion (Figure 4K). Subsequent RNA pull‑down experiments confirmed that motif mut1 mediated the interaction between SRSF1 and PML pre‑mRNA (Figure 4L).

To test whether the predicted acetylation site affects PML splicing and SRSF1 binding, we generated PML wild‑type (WT) and mutant (MUT) PML minigenes (Figure S5E). In NAT10‐overexpressing ADSCs, the WT minigenes showed efficient exon 5 skipping, whereas the MUT minigenes exhibited exon 5 retention (Figure S5F). RNA pull‑down assays confirmed that SRSF1 bound to PML‐WT but not to PML‐MUT (Figure S5G). Thus, acetylation of this specific cytidine on PML pre‑mRNA is essential for NAT10‑mediated splicing regulation and SRSF1 recruitment. Collectively, these data indicate that SRSF1, recruited by NAT10 through ac4C modification, promotes PML exon skipping by recognising the splice site of PML pre‐mRNA (Figure 4M).

### 3.5 NAT10 promotes SRSF1‐dependent PML exon skipping and reduces PCBP1‐mediated PML exon inclusion

**FIGURE 5:**
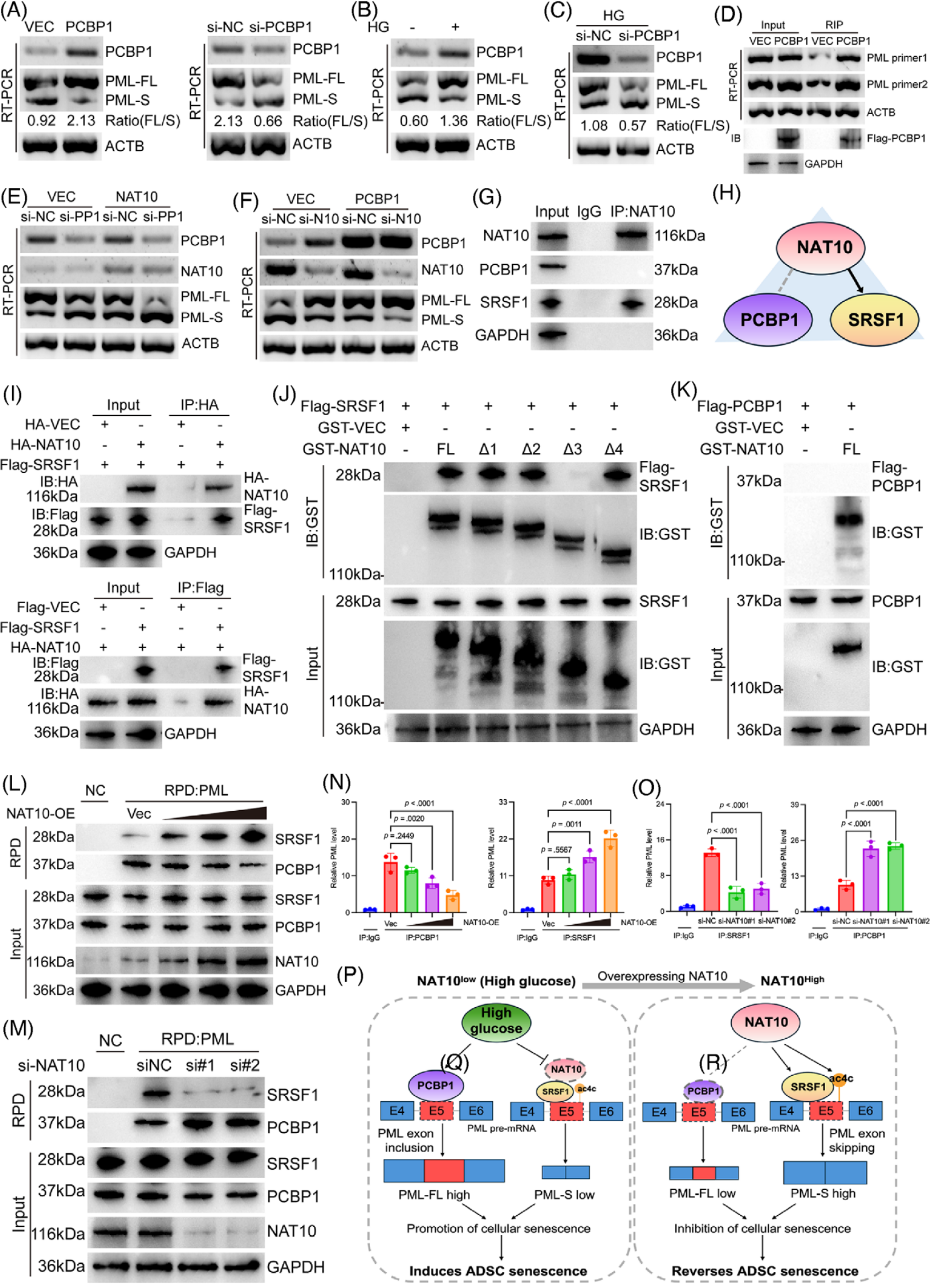
N‐acetyltransferase 10 (NAT10) disrupts PCBP1‐mediated PML exon inclusion and promotes SRSF1‐dependent exon skipping of PML. (A) RT‑PCR of PML splicing after PCBP1 overexpression or knockdown (transfected with si‐PCBP1). (B) RT‑PCR of PCBP1 expression and PML variants under high glucose (HG). (C) RT‑PCR of PML splicing after si‑PCBP1 under HG. (D) RIP‑qPCR showing PCBP1 binding to PML pre‑mRNA. (E) RT‐PCR of PML splicing in adipose‐derived stem cells (ADSCs) with PCBP1 knockdown and NAT10 overexpression. (F) RT‐PCR of PML splicing in ADSCs with PCBP1 overexpressed and NAT10 depletion. (G) Co‑immunoprecipitation (co‑IP) of endogenous NAT10, PCBP1 and SRSF1. (H) Schematic diagram of the relationship of NAT10, PCBP1 and SRSF1. (I) Co‐IP of exogenous HA‐NAT10 and Flag‐SRSF1 in HEK‐293T cells. (J, K) GST pull‑down assays mapping the NAT10 domain interacting with SRSF1 (J) and showing no direct binding to PCBP1 (K). (L, M) RNA pull‑down assessing PCBP1 and SRSF1 binding to PML mRNA after NAT10 overexpression (NAT10‑OE) (L) or si‑NAT10 (M). (N, O) RIP‑qPCR quantifying PCBP1 and SRSF1 binding to PML mRNA after NAT10‑OE (N) or si‑NAT10 (O). (P) Proposed molecular mechanisms of NAT10 action in the regulation of RNA splicing in senescence ADSCs under HG conditions. Data are shown as means ± SD (*n* = 3). *p* values were calculated using one‑way ANOVA with Dunnett post hoc test. Exact *p* values are indicated in the graphs.

PCBP1 has been identified as a splicing factor that directly binds PML pre‑mRNA to modulate its AS.^49^ However, the role of PCBP1 in regulating aberrant RNA splicing in ADSC remains unclear. To verify this, we first overexpressed and knocked down PCBP1 in ADSCs. Our results demonstrated that PCBP1 promoted PML exon 5 inclusion (Figures 5A and S6A,B). RT‐PCR analysis revealed that PCBP1 expression was increased, and that the PML‐FL/S ratio was elevated in ADSCs under HG conditions. In addition, PCBP1 depletion significantly reduced the PML‐FL/S ratio in ADSCs under HG conditions (Figures 5B,C and S6C,D). RIP assays further showed that PCBP1 protein bound PML pre‐mRNA (Figure 5D).

We then found that overexpression of NAT10 in PCBP1‐depleted ADSCs further promoted PML exon skipping induced by PCBP1 downregulation (Figures 5E and S6E). Similarly, NAT10 suppression in PCBP1‐overexpressing ADSCs further enhanced PCBP1‐induced PML exon inclusion (Figures 5F and S6F).

To dissect how NAT10 modulates PCBP1/SRSF1‑dependent PML splicing, we examined the interaction between NAT10 and the PCBP1/SRSF1–PML complex. co‑IP assays revealed that NAT10 interacted with SRSF1, but not with PCBP1, in ADSCs (Figure 5G,H). Reciprocal co‑IP of expressed HA/Flag‑tagged NAT10 and Flag/HA‑tagged SRSF1 further confirmed this interaction (Figure 5I). We constructed GST‑tagged NAT10 domain‐deletion mutants to identify the region responsible for SRSF1 binding (Figure S6G).^16^ GST pull‐down assays revealed that only NAT10‐Δ3, a mutant lacking the GNAT domain, failed to bind SRSF1, whereas other truncation mutants retained binding activity, suggesting that SRSF1 interacts with the NAT10 GNAT domain (Figure 5J). Moreover, GST pull‐down assays demonstrated that NAT10 did not directly bind to PCBP1 (Figure 5K).

Further analysis showed that NAT10 overexpression enhanced the binding of SRSF1 to PML mRNA in a dose‐dependent manner, while simultaneously inhibiting the interaction between PCBP1 protein and PML mRNA (Figure 5L,M). Conversely, NAT10 suppression significantly inhibited the interaction between SRSF1 and PML mRNA and promoted the binding of PCBP1 to PML mRNA (Figure 5N,O). These results suggest that NAT10 recruits SRSF1 and reduces PCBP1 binding to PML, thereby promoting PML exon skipping (Figure 5P).

### 3.6 NAT10 shifts PML splicing towards PML‐S and enhances the therapeutic potential of ADSCs in diabetic wound repair

**FIGURE 6:**
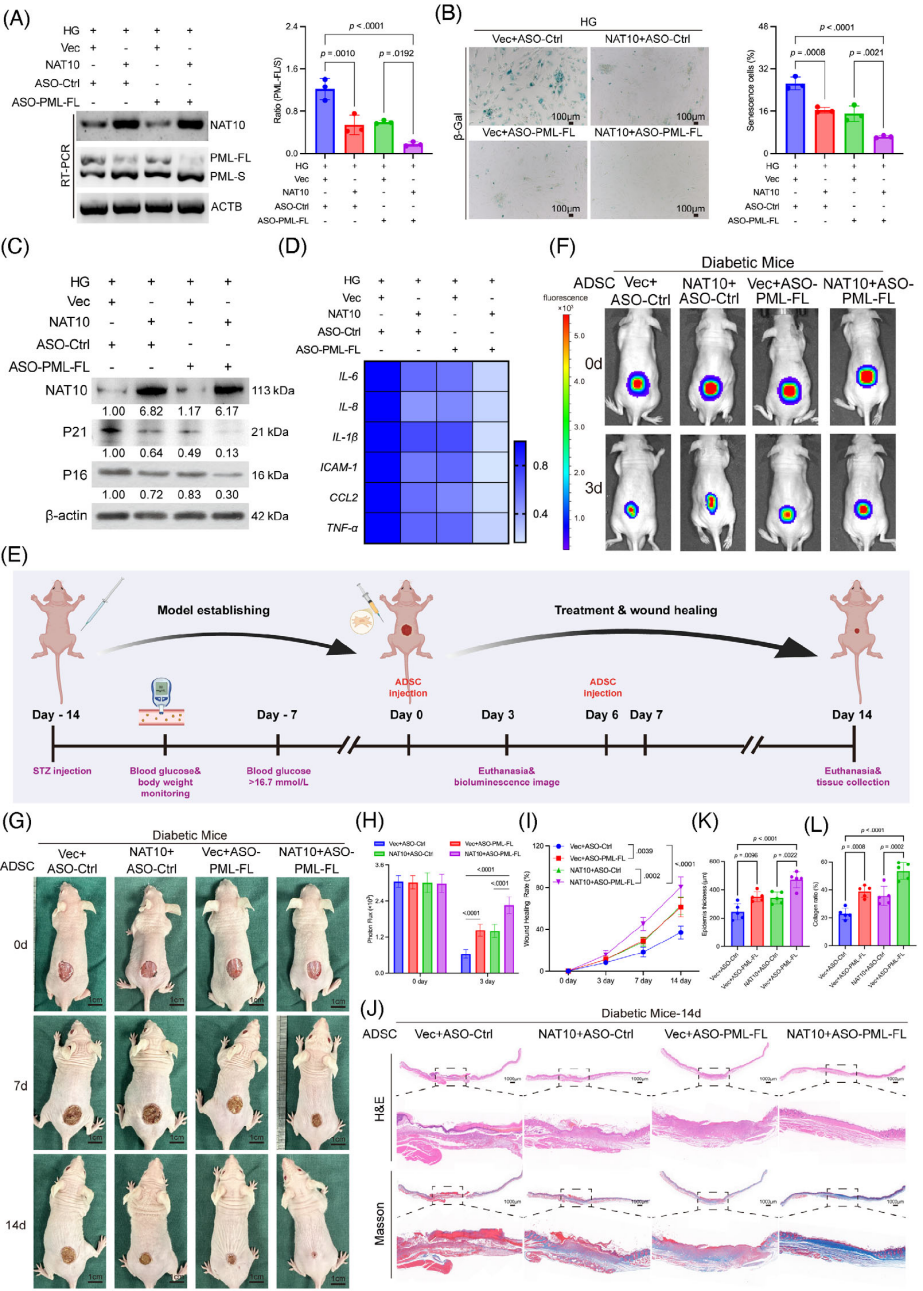
NAT10 shifts PML splicing towards PML‐S, reversing adipose‐derived stem cell (ADSC) senescence and enhancing diabetic wound healing. (A) RT‑PCR of PML splicing in ADSCs transfected with vector (Vec), NAT10‐OE, ASO‐control and ASO‐PML‐FL under high glucose (HG). (B) β‐Gal staining of ADSCs. Scale bar, 100 µm. (C) Western blot of NAT10, p21 and p16 in ADSCs. (D) RT‐qPCR analysis of senescence‐associated secretory phenotype (SASP) genes in ADSCs. (E) Schematic of in vivo experimental protocol. (F, H) ADSCs were infected with NAT10‐OE vector and control vectors, as well as ASO‐control and ASO‐PML‐FL and subsequently seeded into chronic wounds of diabetic mice. (F) Distribution of CM‐Dil‐labelled ADSCs in wounds. (G) Gross wound appearance at Days 0, 7 and 14. Scale bar, 1 cm. (H) Quantification of fluorescence in the images. (I) Quantification of wound healing rate. (J) Hematoxylin and eosin (H&E) and Masson staining at Day 14. Scale bar, 1000 µm. (K) Quantification of epidermis thickness in each group at Day 14. (L) Quantification of collagen deposition in each group at Day 14. Data are shown as means ± SD (*n* = 3 for in vitro; *n* = 5 mice per group for in vivo). *p* values were calculated using one‑way ANOVA with Bonferroni post hoc test. Exact *p* values are indicated in the graphs.

To define the role of NAT10‑mediated PML splicing in the regenerative function of ADSCs in diabetic mice, we constructed ASOs to selectively reduce PML‐FL expression and transfected ADSCs with a NAT10 overexpression plasmid to enhance NAT10 expression. RT‐PCR showed that NAT10 overexpression and PML‐FL depletion significantly reduced the PML‐FL/PML‐S ratio (Figure 6A). In vitro, NAT10 overexpression and PML‐FL depletion reduced ADSC senescence (Figures 6B–D and S7A,B).

Subsequently, we injected these ADSCs into the wounds of diabetic mice and assessed ADSC survival and wound healing outcomes (Figure 6E). NAT10‐overexpressing ADSCs exhibited increased survival in the wound bed compared with vector‐transfected ADSCs, and this effect was further enhanced by PML‐FL inhibition (Figure 6F,G). Furthermore, both PML‐FL suppression and NAT10 overexpression enhanced the reparative capacity of ADSCs (Figure 6H,I). In addition, ADSCs with NAT10 overexpression and PML‐FL depletion promoted skin regeneration and increased collagen fibre deposition in diabetic wounds (Figure 6J–L). These results demonstrate that NAT10 shifts PML splicing from PML‐FL towards PML‐S, thereby reducing ADSC senescence and enhancing their therapeutic efficacy in diabetic wound healing.

## 4 DISCUSSION

**FIGURE 7:**
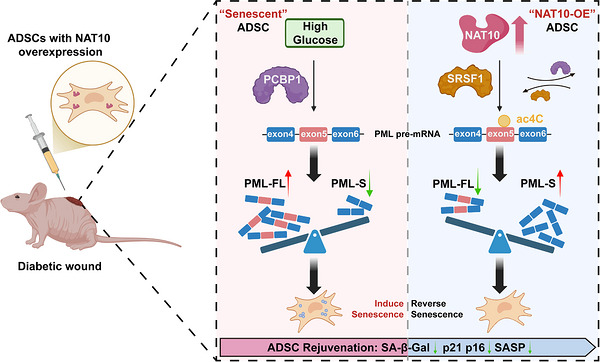
Schematic diagram of the mechanisms. N‐acetyltransferase 10 (NAT10) catalyses ac4C modification on PML pre‐mRNA, which facilitates SRSF1 binding and promotes exon 5 skipping, increasing the protective PML‑S isoform. Concurrently, NAT10 disrupts PCBP1 interaction with PML pre‑mRNA, reducing the pro‑senescence PML‑FL isoform. This shift in PML splicing alleviates adipose‐derived stem cell (ADSC) senescence and enhances their repair capacity, thereby accelerating diabetic wound healing (Image created with BioRender).

Despite the therapeutic potential of stem cell‑based approaches in promoting wound closure, the HG microenvironment of diabetic wounds induces ADSC senescence and compromises their regenerative efficacy. Accordingly, enhancing the regenerative capacity of ADSCs has become an important focus in diabetic wound treatment. This study uncovers a novel role of NAT10 in ADSCs, showing that NAT10 regulates the splicing of the senescence‐related gene PML, thereby reversing ADSC senescence and improving diabetic wound healing (Figure 7). PML‐FL and PML‐S, generated by AS of PML pre‐mRNA, exert opposing biological effects: PML‐FL promotes cellular senescence, whereas PML‐S suppresses senescence. Mechanistically, NAT10 promotes SRSF1 binding to PML pre‐mRNA while reducing PCBP1 binding to PML, thereby inhibiting PCBP1‐mediated exon inclusion and facilitating SRSF1‐modulated exon skipping. Furthermore, combining NAT10 overexpression with PML‐FL inhibition modulates PML splicing and significantly enhances therapeutic efficacy in diabetic wound healing.

ADSC‑based therapy frequently shows limited efficacy in diabetic wound repair, likely because of glucolipotoxicity, oxidative stress and hypoxia within the wound bed, all of which contribute to ADSC senescence and reduce cell viability.^50^, ^51^, ^52^ Consistent with previous reports,^39^, ^53^, ^54^ we found that HG exposure increased ADSC senescence, elevated p21 and p16 protein levels, and upregulated SASP expression (Figure S1). These findings prompted us to explore strategies for alleviating ADSC senescence.

NAT10 is the sole known eukaryotic enzyme responsible for catalysing ac4C modification on RNA and also exhibits protein acetyltransferase activity. This modification participates in diverse biological processes, including stem cell fate regulation, cellular senescence and DNA damage repair.^40^, ^41^ For instance, NAT10‑dependent ac4C modification stabilises RUNX2 mRNA, which in turn facilitates osteogenic differentiation of bone marrow mesenchymal stem cells and alleviates aging‑related osteoporosis.^55^ Ling et al. reported that increased NAT10 expression enhances cellular resistance to H_2_O_2_‐induced senescence.^56^ Although NAT10 has been linked to the control of cellular senescence and stem cell function, its potential role in ADSC senescence remained unexplored. Here, we found that NAT10 overexpression attenuated HG‑induced ADSC senescence and improved the therapeutic outcomes of ADSCs in diabetic wound healing.

While ac4C modification is recognised for its roles in stabilising mRNAs and promoting translation,^57^, ^58^ our findings indicate that NAT10‑mediated ac4C also contributes to RNA splicing, a function that has recently begun to be explored.^24^, ^25^, ^59^ Consistent with this, RNA splicing‐related pathways were highly enriched among NAT10‐interacting proteins by GO analysis, suggesting that NAT10‐mediated regulation of RNA splicing may represent a key molecular mechanism underlying ADSC senescence. RNA‑seq analysis identified PML as a splicing target of NAT10 in ADSCs. NAT10 bound and acetylated PML pre‐mRNA, and an ac4C‐binding‐deficient NAT10 mutant failed to rescue NAT10‐knockdown‐mediated PML exon inclusion, indicating that ac4C modification is required for this splicing event. Direct mutation of the predicted acetylated cytidine on PML pre‐mRNA likewise abolished exon 5 skipping and SRSF1 recruitment, indicating that ac4C modification at this specific site is a prerequisite for the splicing switch. Furthermore, NAT10 recruited SRSF1 to promote PML exon skipping. Minigene reporters identified one of two predicted SRSF1‐binding sites as essential for exon skipping, and RNA pull‐down confirmed that SRSF1 binding depends on this motif. Further validation of this direct interaction by EMSA would provide independent biochemical evidence for SRSF1 binding to this motif. Under HG or NAT10‐deficient conditions, PCBP1 bound to PML pre‐mRNA and promoted splicing towards the PML‐FL isoform. In contrast, NAT10 overexpression enhanced SRSF1 binding to PML pre‐mRNA, while concomitantly reducing PCBP1 occupancy. Given that NAT10 did not directly interact with PCBP1 in co‐IP and GST pull‐down assays, this reciprocal binding pattern likely reflects an indirect mechanism: NAT10‐mediated ac4C modification and SRSF1 recruitment may remodel the RNA‐protein complex, thereby decreasing PCBP1 binding rather than direct displacement. Although the current data support this indirect exchange model, the precise molecular details of how NAT10 ultimately reduces PCBP1 occupancy remain to be fully elucidated. Future studies will dissect this RNA–protein complex remodelling process and its role in AS. Together, these findings indicate that ac4C modification is essential for NAT10‐mediated splicing of the senescence‐related gene PML.

The PML pre‐mRNA consists of nine exons and undergoes AS to generate multiple isoforms with distinct biological functions.^34^ PML exhibits a complex role in cellular senescence. For instance, PML silencing induces cellular senescence in triple‐negative breast cancer cells and inhibits excessive tumour growth in vivo.^32^ Conversely, PML overexpression induces p53‐dependent senescence in both human and murine fibroblasts.^33^, ^45^ These apparently divergent functions may be explained, at least in part, by the generation of distinct PML isoforms with different biological activities. Our data indicate that NAT10 promotes exon 5 skipping of PML pre‑mRNA, shifting the balance towards PML‑S and reducing PML‑FL. In vitro and in vivo experiments subsequently demonstrated that PML‑S overexpression markedly suppressed cellular senescence and enhanced the regenerative capacity of ADSCs. Collectively, our findings identify a new senescence‐associated splicing event and demonstrate that PML‐S and PML‐FL exert opposing effects on ADSC senescence.

In summary, we identified the ac4C writer NAT10 as a critical protector of senescent ADSC and uncovered its novel role in regulating RNA splicing. This work elucidates the relationship between RNA acetylation and AS and uncovers a previously unrecognised regulatory mechanism of cellular senescence. Beyond reversing senescence, this NAT10‑mediated splicing switch may enhance the metabolic adaptability and stress resilience of ADSCs within the diabetic wound microenvironment. By promoting PML‑S production, NAT10 may help ADSCs resist HG, oxidative stress, hypoxia and inflammatory cytokines, thereby improving their engraftment, paracrine function and therapeutic efficacy. These findings suggest that targeting NAT10 or PML splicing may improve ADSC‐based therapies for diabetic wounds. Notably, combining NAT10 overexpression with PML‐FL inhibition further enhanced wound healing, providing a potential therapeutic strategy for diabetic wound treatment.

Regarding cellular sources, the ADSCs used in this study were isolated from 10 independent female donors. In each independent experiment, cells from a single donor were used, and experiments were repeated using cells from different donors to ensure reproducibility while minimising inter‑donor variability within individual experiments. Nevertheless, donor‑to‑donor variability in NAT10 expression, ac4C modification or splicing regulation cannot be completely excluded. Future studies involving larger donor cohorts and single‑cell transcriptomic analysis could further clarify how inter‐individual heterogeneity influences therapeutic outcomes.

Although our study provides strong evidence for the NAT10–ac4C–SRSF1 axis in PML splicing and ADSC senescence, several mechanistic questions remain unresolved. First, the molecular details of the indirect exchange between SRSF1 and PCBP1 on PML pre‑mRNA remain to be fully defined. Second, although the SRSF1‐PML pre‐mRNA interaction has been validated by minigene reporters and RNA pull‐down, direct confirmation of this binding by EMSA will be examined in future studies. Third, we showed that modulating NAT10 and PML‐FL promotes wound healing; however, whether these effects are mediated by the splicing factors requires further testing by complete rescue experiments. Overexpressing or depleting these splicing factors in the context of NAT10 overexpression or knockdown would verify their functional necessity in ADSC senescence and wound repair. Fourth, while we used siRNA and a chemical inhibitor to validate NAT10 specificity, these approaches have inherent limitations, including off‑target effects and incomplete inhibition. Future studies using NAT10‑knockout ADSCs or mouse models will be essential to provide definitive validation of NAT10's physiological and therapeutic roles. Fifth, the upstream regulators of NAT10 expression or activity in the diabetic microenvironment remain unknown, and additional downstream effectors beyond PML may also contribute to its anti‑senescence effects. Addressing these questions in future studies will help optimise ADSC‑based therapies for diabetic wound healing and may uncover new therapeutic targets.

Overall, our findings reveal a novel mechanism linking RNA acetylation to AS in stem cell senescence and suggest that modulation of NAT10 or PML splicing may represent a promising strategy to improve ADSC‑based therapies for diabetic wounds.

## AUTHOR CONTRIBUTIONS

Wuhan Wei, Chanyuan Jiang and Dong Zhu conceived and designed the study. Wuhan Wei, Chanyuan Jiang, Xuefeng Han, Xuan Ma, Xinyu Jia, Jiaqi Ling and Rui Zhang performed the experiments and analysed the data. Facheng Li and Ningbei Yin supervised the study and provided critical resources. Wuhan Wei wrote the original draft. All the authors reviewed and approved the final manuscript.

## CONFLICT OF INTEREST STATEMENT

The authors declare no conflicts of interest.

## ETHICS STATEMENT

This study was conducted in accordance with the Declaration of Helsinki. The collection and utilisation of adipose tissues were approved by the Ethics Committee of Plastic Surgery Hospital, Chinese Academy of Medical Sciences (Approval No. EAEC 2021‐I2M‐1‐052). All animal experiments were approved by the Animal Care and Ethics Committee of Plastic Surgery Hospital, Chinese Academy of Medical Sciences (Project No. EAEC 2025‐024).

## Supporting information



Supporting Information

Supporting Information

Supporting Information

Supporting Information

Supporting Information

Supporting Information

Supporting Information

Supporting Information

## Data Availability

The RNA‐seq data generated in this study have been deposited in the Gene Expression Omnibus (GEO) under accession number GSE326590. All other data supporting the findings of this study are available from the corresponding author upon reasonable request.
