# Supplementary figures and images for: NAT10‐mediated N4‐acetylcytidine modification drives RNA splicing of PML to alleviate adipose‐derived stem cell senescence and promote diabetic wound healing
Source: Clin Transl Med. 2026 Jun 11;16(6):e70711. doi: 10.1002/ctm2.70711 (PMC13260675; doi:10.1002/ctm2.70711)

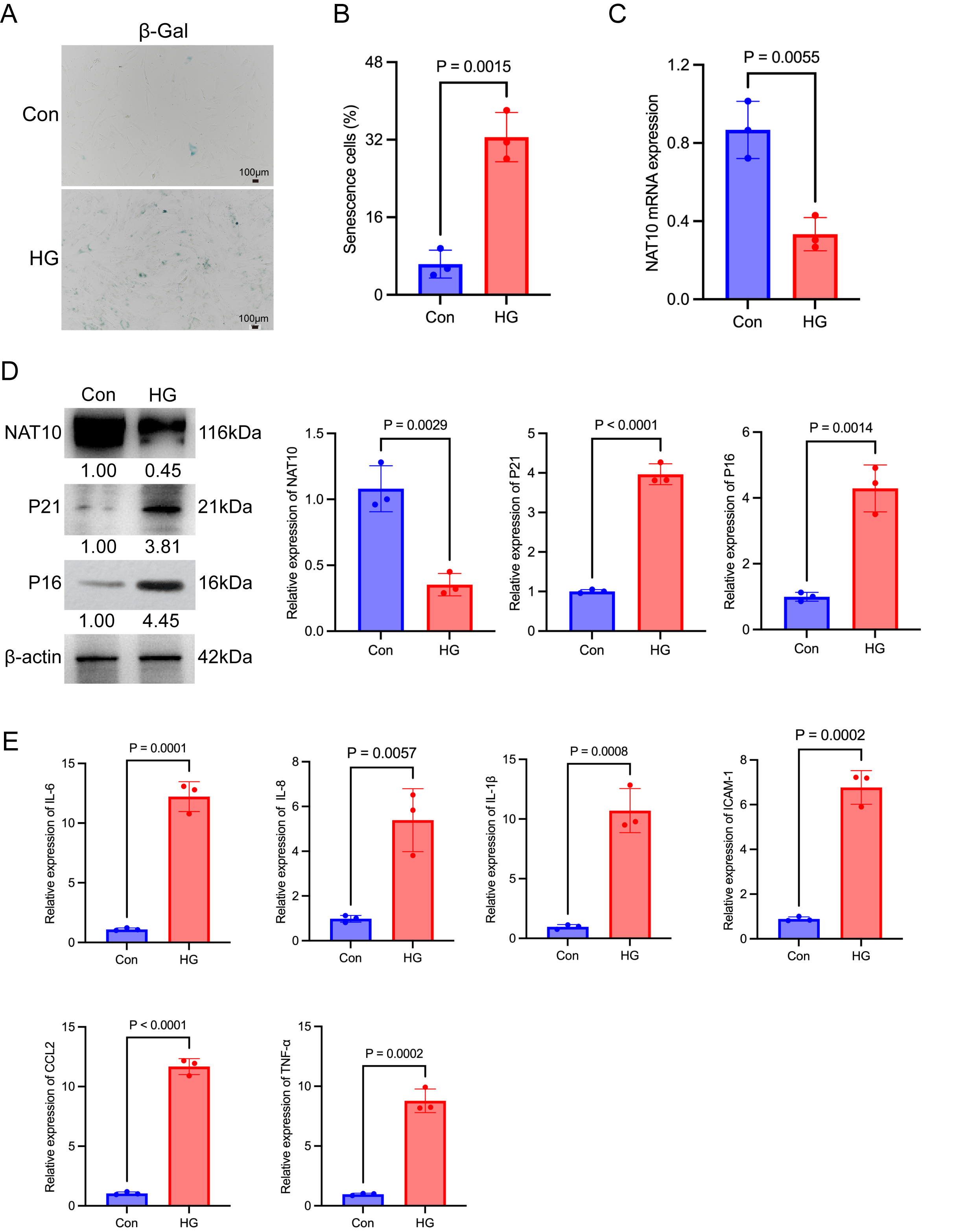

Supplement: Supplementary file 1 — Supporting Information [file CTM2-16-e70711-s002.tif]

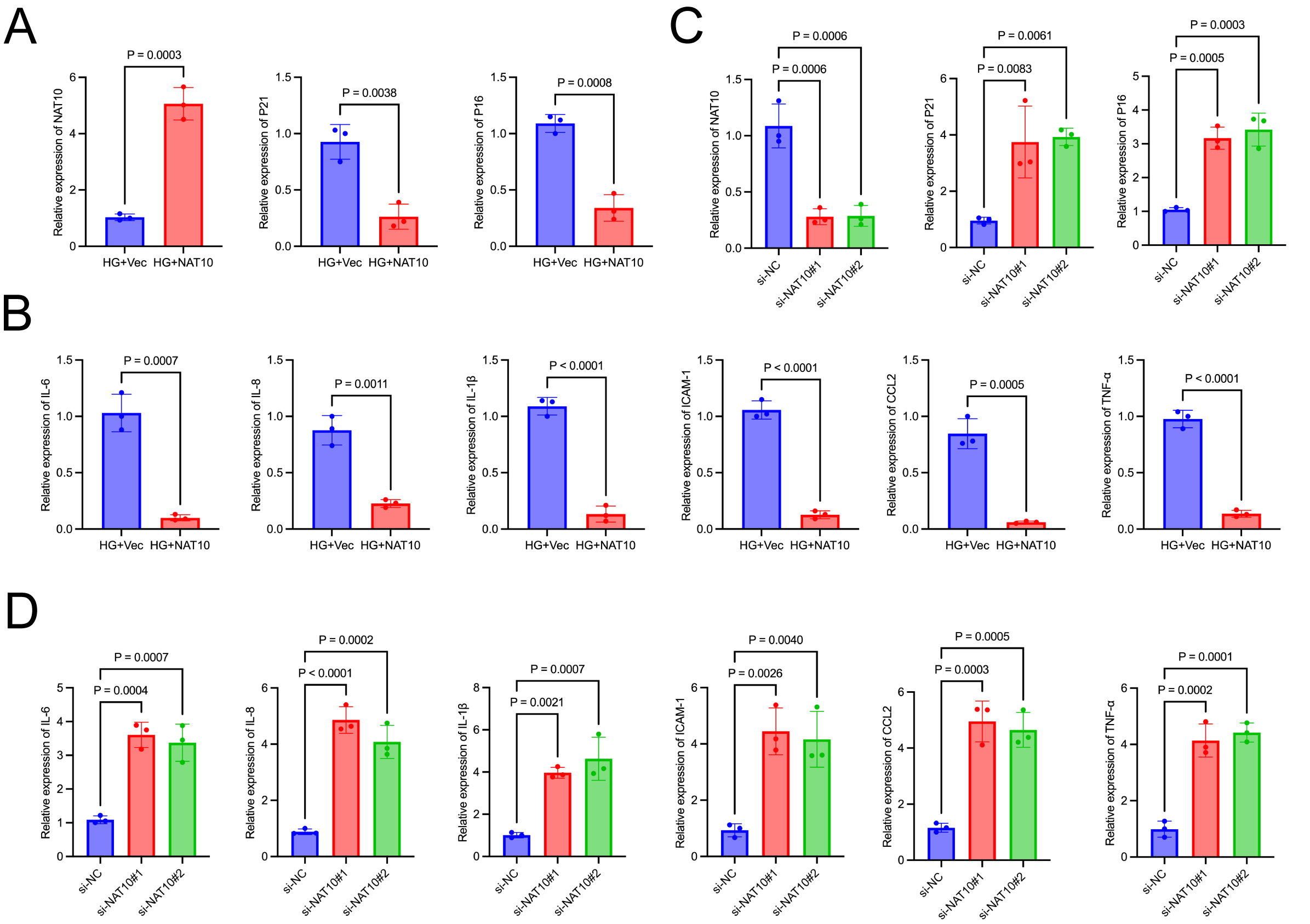

Supplement: Supplementary file 2 — Supporting Information [file CTM2-16-e70711-s001.tif]

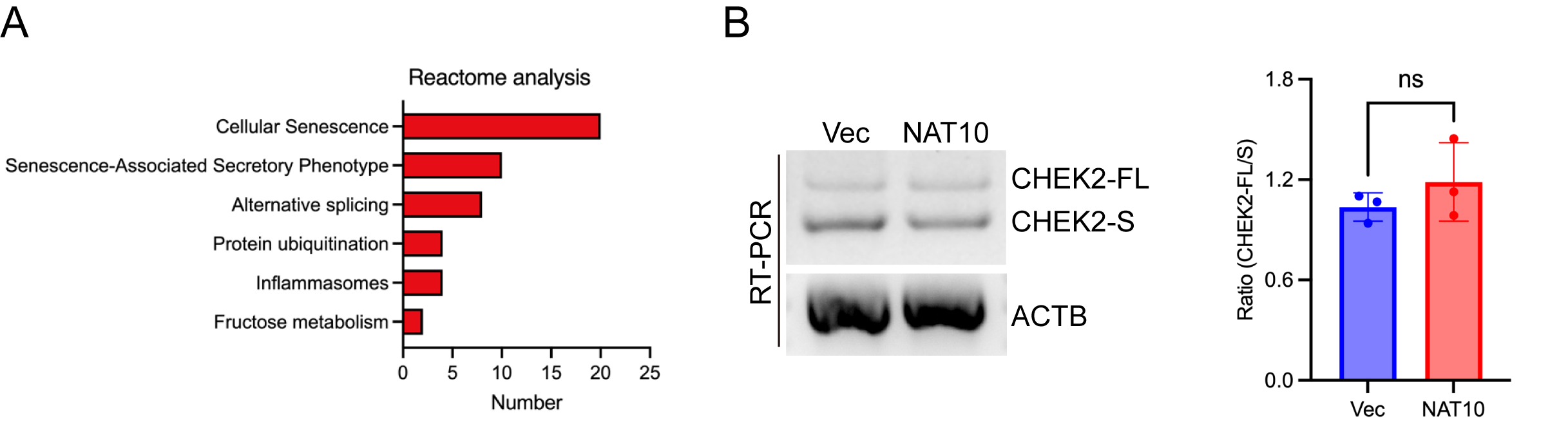

Supplement: Supplementary file 3 — Supporting Information [file CTM2-16-e70711-s004.tif]

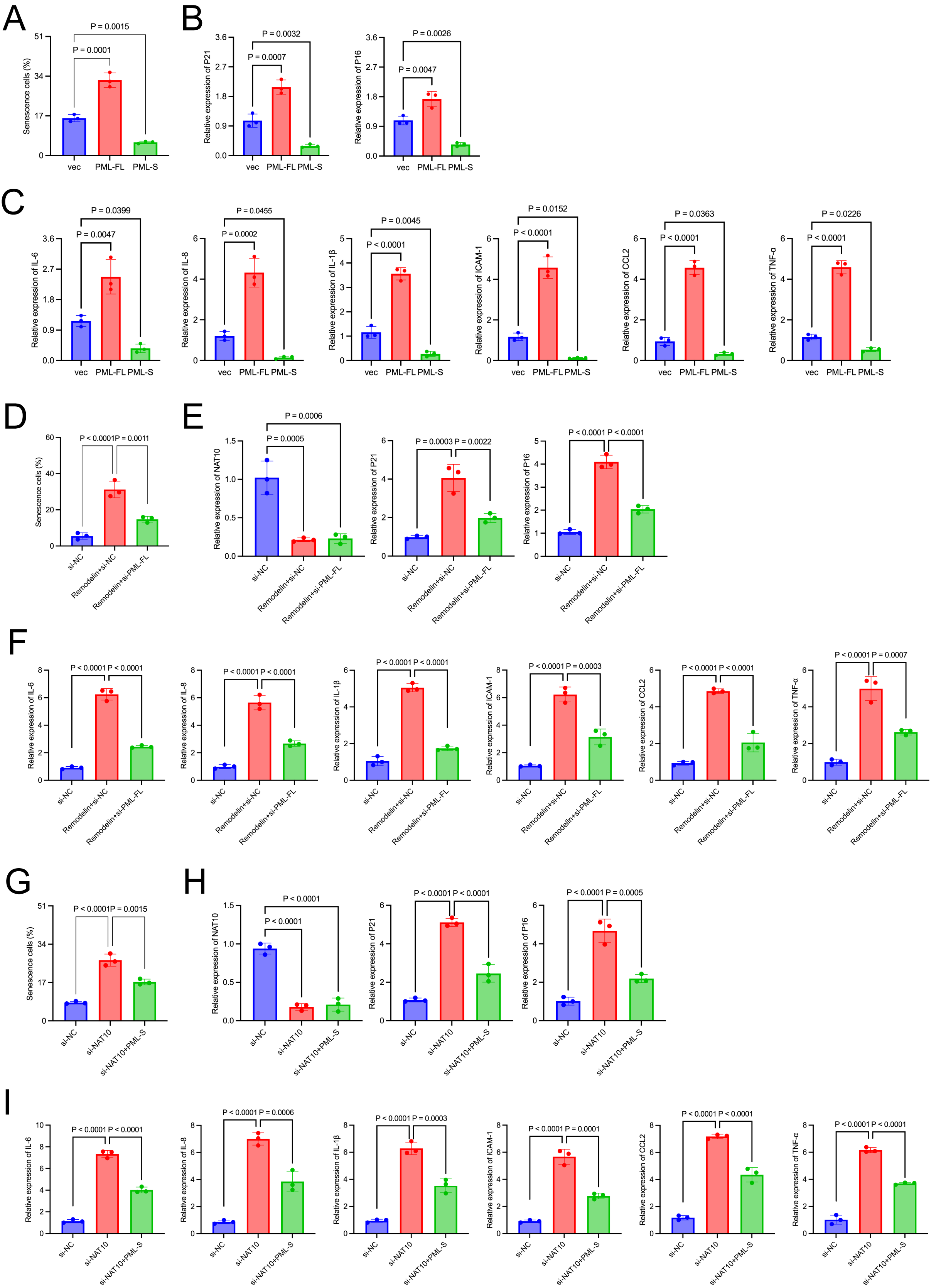

Supplement: Supplementary file 4 — Supporting Information [file CTM2-16-e70711-s007.tif]

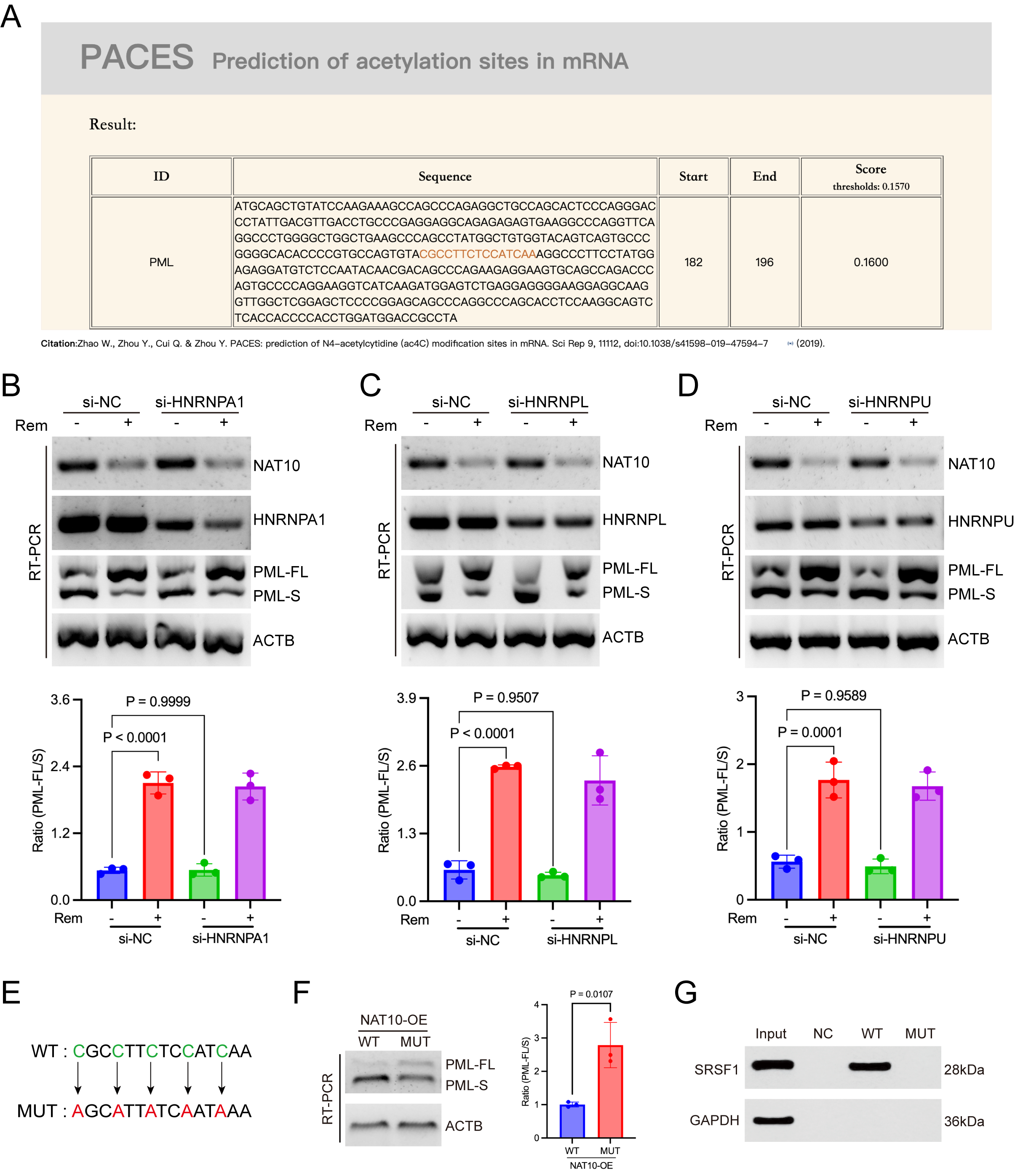

Supplement: Supplementary file 5 — Supporting Information [file CTM2-16-e70711-s005.tif]

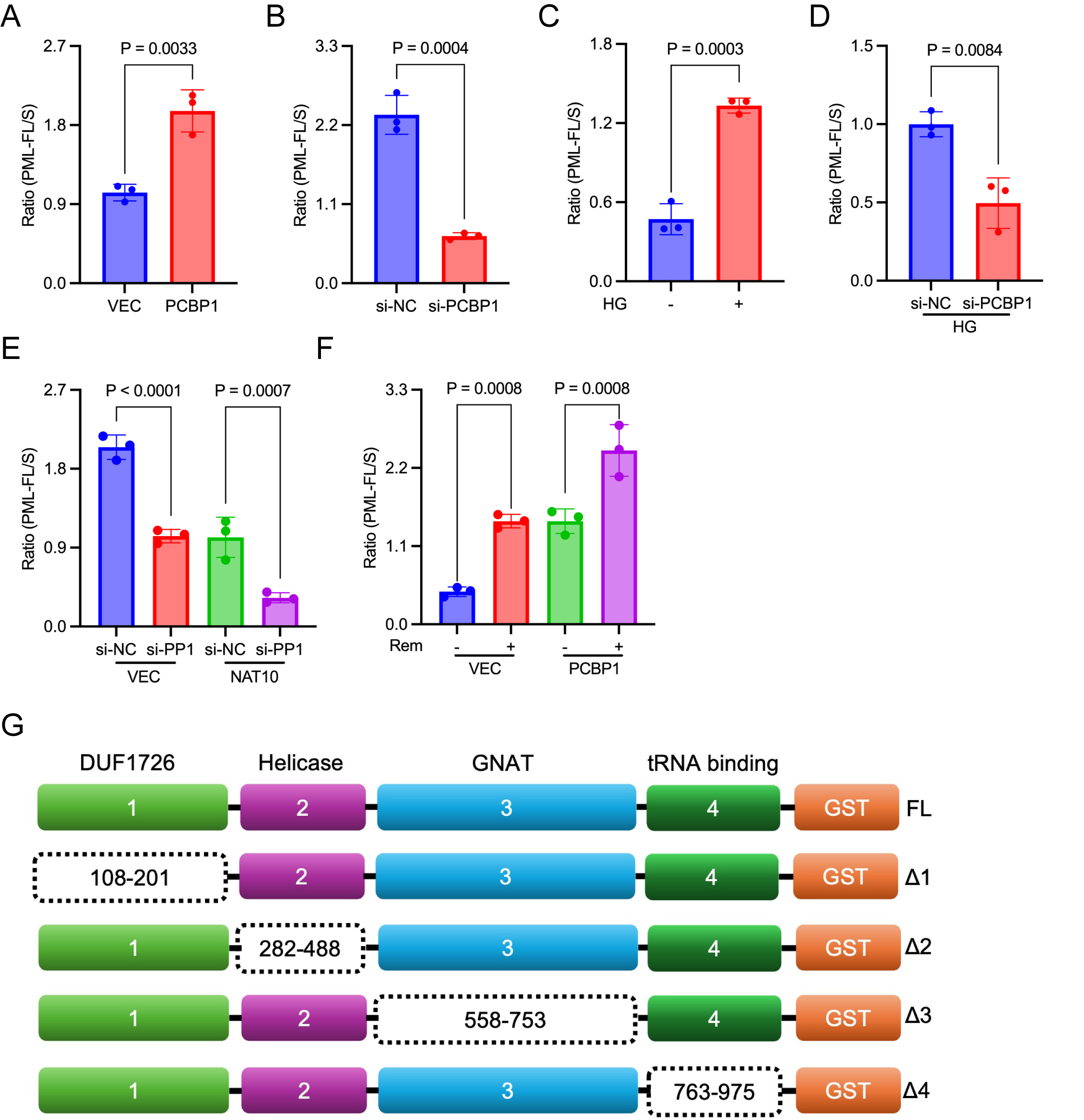

Supplement: Supplementary file 6 — Supporting Information [file CTM2-16-e70711-s006.tif]

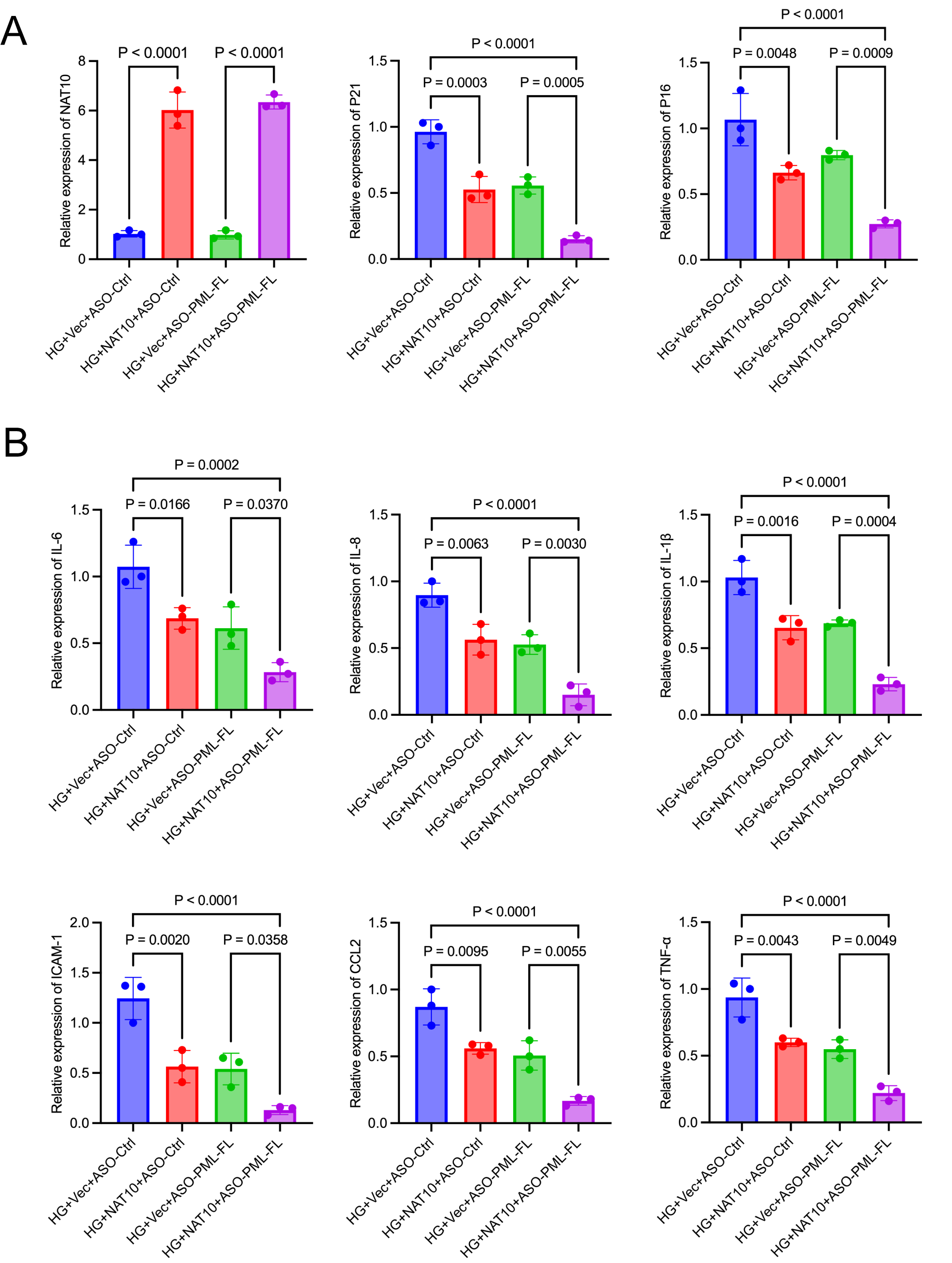

Supplement: Supplementary file 7 — Supporting Information [file CTM2-16-e70711-s003.tif]

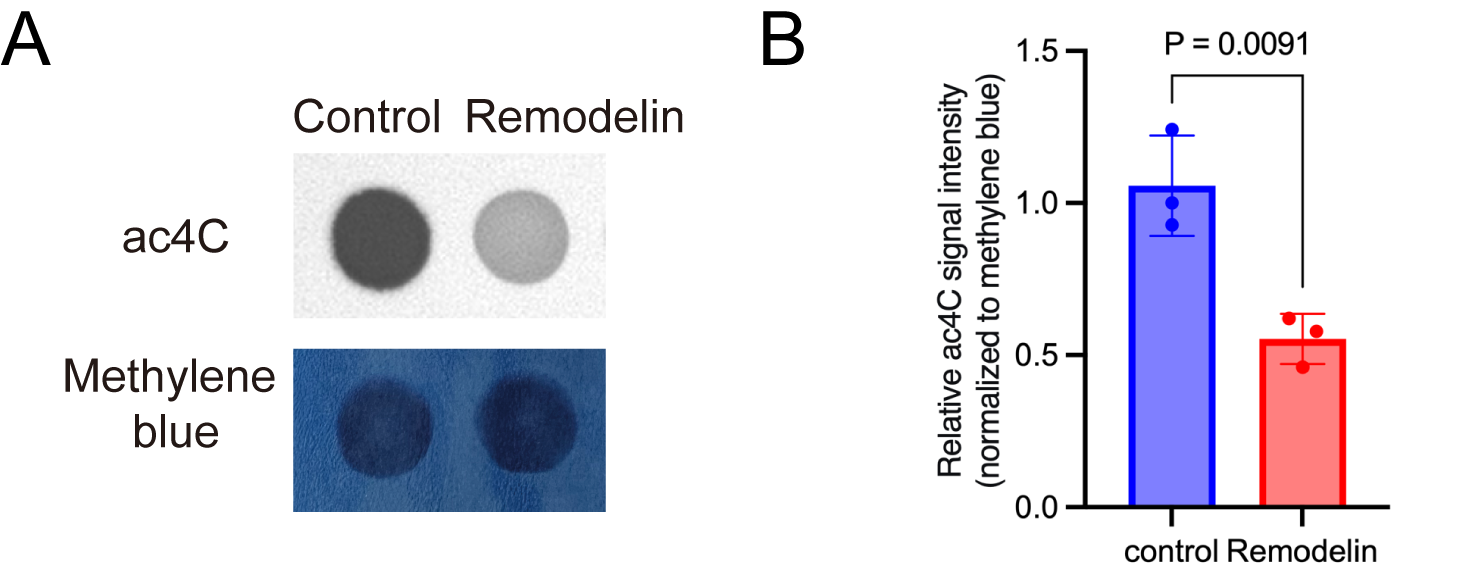

Supplement: Supplementary file 8 — Supporting Information [file CTM2-16-e70711-s008.tif]
